# Supplementary material for: Exploring the association between sick child healthcare utilisation and health facility quality in Malawi: a cross-sectional study
Source: BMJ Open. 2019 Jul 27;9(7):e029631. doi: 10.1136/bmjopen-2019-029631 (PMC6661667; doi:10.1136/bmjopen-2019-029631)
Supplement: Supplementary data [file bmjopen-2019-029631supp001.pdf]

Appendix Table 1: Completeness of indicators for facilities† (n=920) and observations

Variable	% Missing	% Completed
Water (improved water source round within 500 meters)	0	100
Ambulance (Functional with fuel)	0	100
Electricity	0	100
Communication (phone or short wave radio)	0	100
Toilet (functional & observed)	0	100
General facility cleanness (floor, table, chairs)	0	100
Client waiting room	0	100
Access to computer with e-mail and Internet	0	100
Light source (functional & observed)	0	100
Sick child health service room infection control	0	100
Sharps (all rooms have sharps box)	0.33	99.67
Medical waste is adequately disposed	0.43	99.57
Functional thermometer in sick child health service area	0	100
Functional stethoscope in sick child health service area	0	100
Oral rehydration salts in pharmacy or sick child health service area	0	100
Amoxicillin tablet or syrup observed and valid	0.65	99.35
Zinc tablet or syrup observed and valid	0.65	99.35
Medications, vaccines stored according to expiration date	11.96	88.04
Adequate storage for medications	0	100
Computer or stock ledger updated daily	0	100
Staff training on child health services	0	100
Last supervisory visit within 6 months	0	100
Management team meeting every 6 months	0.65	99.35
Supervisory used checklist for quality of health services data	13.70	86.30

Supervisory: facility performance	13.70	86.30
Supervisory: helped facility make data-based decision	13.70	86.30
Supervisory: provided feedback	13.70	86.30
Reporting client opinion in place	1.20	98.8
Routine quality assurance activities	0.87	99.13
Asked about vomiting	18.91	81.09
Asked about unable to drink	18.91	81.09
Asked about convulsions	18.91	81.09
Asked about normal feeding when not ill	18.91	81.09
Asked about maternal HIV status	18.91	81.09
Asked about feeding during illness	18.91	81.09
Asked about fever	18.91	81.09
Asked about ear pain	18.91	81.09
Asked about diarrhea	18.91	81.09
Asked about coughing	18.91	81.09
Examination: weighed	18.91	81.09
Examination: took temperature	18.91	81.09
Examination: checked palms/conjunctiva/mouths	18.91	81.09
Examination: checked for edema	18.91	81.09
Counseling: provider explained dosing/medications	19.24	80.76
Counseling: counseled food feeding with providers	18.91	81.09
Counseling: caretaker was told child's diagnosis	18.91	81.09
Counseling: provider described danger signs return to facility	18.91	81.09

[†] A total of 920 health facilities from 2013 Malawi SPA that provided child curative care was included in this study.

Appendix Table 2. Sensitivity analyses model results for the association between sick child healthcare utilization and the overall quality (structural and process quality) of health service facility in Malawi.

**Model**	**Adjusted results** †		**N**
	Odds Ratio (P-value)	95% CI	
Main Model: the single nearest facility	1.66 (0.03)	(1.04, 2.63)	9,695
Sensitivity Analysis 1: the best facility within 5 km	1.88 (0.05)	(0.99, 3.58)	6,429
Sensitivity Analysis 2: the best facility within 10 km	1.81 (0.03)	(1.06, 3.09)	9,296
Sensitivity Analysis 3: the best facility within 20 km	1.85 (0.10)	(0.87, 3.93)	9,682

*** p<.01, ** p<.05, * p<.1

†Adjusted for: sick child age, sex, type of illness, mother education, household wealth quintile, and the household's rural residence.
