# Supplementary material for: Exploring the association between sick child healthcare utilisation and health facility quality in Malawi: a cross-sectional study
Source: BMJ Open. 2019 Jul 27;9(7):e029631. doi: 10.1136/bmjopen-2019-029631 (PMC6661667; doi:10.1136/bmjopen-2019-029631)
Supplement: Author's manuscript [file bmjopen-2019-029631.draft_revisions.pdf]

BMJ Open is committed to open peer review. As part of this commitment we make the peer review history of every article we publish publicly available.

When an article is published we post the peer reviewers' comments and the authors' responses online. We also post the versions of the paper that were used during peer review. These are the versions that the peer review comments apply to.

The versions of the paper that follow are the versions that were submitted during the peer review process. They are not the versions of record or the final published versions. They should not be cited or distributed as the published version of this manuscript.

BMJ Open is an open access journal and the full, final, typeset and author-corrected version of record of the manuscript is available on our site with no access controls, subscription charges or pay-per-view fees (http://bmjopen.bmj.com).

If you have any questions on BMJ Open's open peer review process please email editorial.bmjopen@bmj.com

BMJ Open

Exploring the association between sick child care utilization and health facility quality in Malawi: A Cross-Sectional Study

Journal:	*BMJ Open*
Manuscript ID	bmjopen-2019-029631
Article Type:	Research
Date Submitted by the Author:	11-Feb-2019

Complete List of Authors:	LIU, LINGRUI; Yale University School of Public Health, Health Policy and Management; Global Health Leadership Initiative Yale University<br>Leslie, Hannah; Harvard TH Chan School of Public Health, Department of Global Health and Population<br>Joshua, Martias; Malawi Ministry of Health<br>Kruk, Margaret; Harvard T.H. Chan School of Public Health , Department of Global Health and Population

Keywords:	Quality in health care < HEALTH SERVICES ADMINISTRATION & MANAGEMENT, Organisation of health services < HEALTH SERVICES ADMINISTRATION & MANAGEMENT, Community child health < PAEDIATRICS, PUBLIC HEALTH

SCHOLARONE™
Manuscripts

Title

Exploring the association between sick child care utilization and health facility quality in Malawi: A Cross-Sectional Study

Authors

Lingrui Liu[1,2], Hannah H. Leslie[3], Martias Joshua[4], Margaret E. Kruk[3]

Authors' Affiliations

[1]Department of Health Policy and Management, Yale School of Public Health, New Haven, CT, USA
[2]Global Health Leadership Initiative, Yale University, New Haven, CT, USA
[3]Department of Global Health and Population, Harvard T.H. Chan School of Public Health, Boston, MA, USA
[4]Malawi Ministry of Health, Lilongwe, Malawi

Corresponding author:

Lingrui Liu
Department of Health Policy and Management
Yale School of Public Health
New Haven, Connecticut 06510, USA
Lingrui.liu@yale.edu

Word Count (excluding title page, abstract, references, figures and tables) : 4818

For peer review only - http://bmjopen.bmj.com/site/about/guidelines.xhtml

ABSTRACT (word count: 297)

Objective: Increasing availability of basic healthcare services in low-and-middle-income countries is not sufficient to meet the Sustainable Development Goal target for child survival in high-mortality settings, where healthcare utilization is often inconsistent and quality of care can be poor. We assessed whether poor quality of sick child care in Malawi is associated with low utilization of sick child care.

Design: We measured two elements of quality of sick child care: facility structural readiness and process of care using data from the 2013 Malawi Service Provision Assessment. Overall quality was defined as the average of these metrics. We extracted demographic data from the 2013 Malawi MDG Endline Survey and linked households to nearby facilities using geocodes. We used logistic regression to examine the association of facility quality with utilization of formal health services for children under five suffering diarrhea, fever, or cough/acute respiratory illness, controlling for demographic and socioeconomic characteristics. We conducted sensitivity analyses, modifying the travel distance and population - facility matching criteria.

Setting and Population: 568 facilities were linked with 9,701 children with recent illness symptoms in Malawi, of whom 69% had been brought to a health facility.

Results: Overall, facilities showed gaps in structural quality (62% readiness) and major deficiencies in process quality (33%), for an overall quality score of 48%. Better facility quality was associated with higher odds of utilization of sick child care services (Adjusted odds ratio [AOR]: 1.56, 95% CI:1.04, 2.28), as was structural quality alone (AOR: 1.42, 95% CI:1.00, 2.01). Sensitivity analyses supported the main finding.

Conclusion: Although Malawi's health facilities for curative child care are widely available, quality and utilization of sick child care services are in short supply. Improving facility quality

For peer review only - http://bmjopen.bmj.com/site/about/guidelines.xhtml

may provide a way to encourage higher utilization of care and thereby decrease preventable childhood morbidity and mortality.

Keywords: health facility quality, sick child care, healthcare utilization, Malawi, sub-Saharan Africa

Strengths and limitations of this study

- Using the spatial geocodes, this study linked the health system (Malawi SPA) and the households (Malawi MDG Endline Survey) datasets to investigate the role of quality in sick child care utilization in Malawi.
- This study relied on internationally endorsed guidelines to define quantifiable quality of care measures.
- Multiple sensitivity analyses confirmed the findings.
- However, matching strategy of linking a sick child to a health facility may not completed reflect actual behavior.

For peer review only

For peer review only - http://bmjopen.bmj.com/site/about/guidelines.xhtml

INTRODUCTION

The global health community has achieved notable gains in the Millennium Development Goals (MDGs) era. However, elimination of preventable and treatable child mortality remains an urgent global health priority in the coming decade (1, 2). Over the past decade under the umbrella goal of universal health coverage (UHC) (3, 4), health policies have focused on the expansion of coverage of essential health interventions and basic services in sub-Saharan Africa (SSA) and other low-income regions. Due to the rising recognition that, without improving quality of care in the health system, improved access to health care alone would not achieve expected health outcomes, the global health community has begun to focus on improvement of health-care quality (5, 6). Large expansions of health facility networks have been attained in SSA and other low-income regions, and yet, utilization of available resources for care of sick children under five remains low, resulting in inadequate care-seeking for children with diarrhea, malaria or pneumonia (7-10). Malawi, a sub-Saharan African country whose government has declared reduction of preventable children-under-five mortality as a national priority, achieved the MDG targets for improved child health (11). However, healthcare utilization for conditions treatable by the health system has been lacking, as data from the most recent Demographic and Health Survey (DHS) 2015 in Malawi shows (12). Although healthcare facility utilization increased steadily from 2000 to 2015, the proportion of sick children under five with symptoms of acute respiratory infection (ARI), fever, and diarrhea who were taken to a health provider for treatment within 48 hours of symptom onset, remained insufficient (51%, 46%, and 66%, respectively) (7). These utilization patterns occur despite wide availability of child health services (including outpatient curative care, child growth monitoring, and child vaccination) in Malawi's health facilities (12), with a median distance to the nearest health facility of 5 km and an estimated

For peer review only - http://bmjopen.bmj.com/site/about/guidelines.xhtml

median traveling time of 75 minutes (13). There is a growing recognition that, besides geographic access, poor quality of care could be a supply-side barrier to UHC, deterring patients from obtaining treatment and influencing family decisions to use or avoid services, which would ultimately impact health outcome gains (14-17).

Although Malawi has achieved substantial improvement in coverage (utilization by those in need) for curative care in children, as estimated from household survey data, quality remains weak (18, 19). Moreover, a recent cross-national study demonstrated that the duration and the content of sick child care service was inadequate (20). Another recent study echoed that care quality in pneumonia diagnosis for a sick child in Malawi is poor, with low guideline adherence to the Integrated Management of Childhood Illness (IMCI) criteria (21). Most existing evaluation studies on the quality of sick child care delivered at health facilities in Malawi have aimed to describe the state of quality but few have tackled the question of whether poor quality dissuades families from using the health system when a child falls ill. In the area of reproductive health, one study suggested a positive association between the perceived quality of reproductive care at the facility last attended and the family utilization of immunization and treatment services for their children at these health facilities, as observed within facilities in Kisumu Municipality in Western Kenya (22). Another recent research from a health intervention program in Ntcheu district in Malawi identified that quality of perinatal care provided is an important predictor of both women's use and satisfaction with such services received (23). Furthermore, earlier literature on bypassing for facility delivery in rural Africa has documented that quality of care is influencing clinic choice (24). Although these studies suggested a positive association between respondent perception of quality and utilization, it would be worthwhile to investigate this relationship at a broader population level (e.g., using national household-representative data), as

For peer review only - http://bmjopen.bmj.com/site/about/guidelines.xhtml

well as through use of the most recent health facility assessment standardized surveys, to explore the relationship.

Policymakers need evidence on what health system attributes (e.g., quality of sick child care) may influence primary care utilization. Malawi is focusing on health sector strategies to improve child health and wellbeing, and has embarked on its second Health Sector Strategic Plan (HSSP 2011-2016), making this an opportune time to seek evidence on the health system factors (e.g., quality) that best promote utilization of sick child health care services. In this paper we examine the association between quality of care in health facilities in Malawi and utilization of sick child care by caregivers. This study is one of the few which we are aware that focuses on the contribution of facility quality to utilization of child care services in high-disease-burden settings (25).

METHODS

Study Sample

Malawi is one of the smallest and most densely-populated countries in sub-Saharan Africa, made up of a predominantly young population, of which almost half is aged 15 years or younger, and more than 20% children under five. With a total population of 17 million and a gross domestic product (GDP) per capita of US$1200 in 2015, approximately 52% of Malawi's population lives below the international poverty line ($1.90 per person per day in 2011 purchasing power parity [PPP] dollars), with more than 80% inhabiting rural regions (26). Health facilities that provide child health services in the formal sector of Malawi include hospitals, health centers, clinics, dispensaries, and health posts.

For peer review only - http://bmjopen.bmj.com/site/about/guidelines.xhtml

The analysis in this research combined data from multiple sources. To obtain information on the facility quality of child health services, we used the Malawi 2013-14 Service Provision Assessment (SPA), a comprehensive census of all formal-sector health facilities conducted by the Demographic and Health Surveys (DHS) Program (12). SPA is a standardized cross-sectional survey of public and private health facilities, which includes a facility-level audit, clinical observation protocols, patient exit interviews, and health care provider interviews. The health facility audit was conducted in each health facility visited using a standardized inventory questionnaire. At least one health worker was interviewed in a sampled health facility. Interviewers also observed a sample of patient-provider clinical care service interaction processes, as well as interviewing patients observed receiving care upon their exit from the health facility. The SPA survey does not report or examine clinical outcomes. In this study, we examined the quality of out-patient sick child curative care delivered at health facilities in Malawi, excluding child vaccination services and child growth monitoring services. We limited our sample of facilities to those facilities which provided outpatient sick child curative care services.

To obtain household information and care-seeking for children under five years of age, we used the Malawi 2013-14 MDG Endline Survey (MES), which was conducted by the National Statistical Office as part of the global Multiple Indicator Cluster Surveys (MICS) program (27). Malawi 2014 MES is a household survey conducted to assess health outcomes among a nationally-representative sample of the population, employing a multi-stage sampling strategy. Enumeration areas (EAs) were sampled within the strata of district and urban versus rural location, and then households were identified within EAs. With a systematic sample of 25 households drawn in each sample cluster, a total sample of 1140 EAs and 28,479 households

For peer review only - http://bmjopen.bmj.com/site/about/guidelines.xhtml

were selected for the Malawi 2014 MES. We limited our sample of households to those in which caregivers reported children under five years of age who had an episode of diarrhea, symptoms of acute respiratory infection (ARI), or fever during the two weeks prior to the survey. Although these three specific medical conditions cannot cover the full range of illnesses of children under five, malaria, diarrhea, and pneumonia remain leading causes of death among children under age five and are the most consistent indicators for child disease burden across the globe, with the vast majority occurring in LMICs (28). These conditions combined are relevant in epidemiological predictions and represent demand for sick child care services in Malawi.

Measure of quality of child health services delivered at health facilities in Malawi

To date, there has not been a single uniform set of measures on quality definition and metrics (15, 29). The Institute of Medicine report *Crossing the Quality Chasm* identified six dimensions to measure quality: safe, effective, patient-centered, timely, efficient, and equitable (30). We consolidated these dimensions with Donabedian's (1980) quality of care framework of structure, process and outcome (31). The structure elements describe the characteristics of the environment in which care is provided, which exist before the care takes place. The structural inputs of a health system indicate the capability and serviceability of a health system under which care occurs (32). Process features include two key components: technical interventions (appropriate delivery of clinical procedures following clinical guidelines, e.g., WHO Integrated Management of Childhood Illness [IMCI] guidelines in child curative care services) and inter-personal interactions between users and a health care system. Outcomes refer to the final consequences of care, such as the under-five mortality rate (32). In this study, available data did not specify patient outcomes for the children experiencing recent illness.

For peer review only - http://bmjopen.bmj.com/site/about/guidelines.xhtml

Using Donabedian's framework, we constructed an index of structural quality based on the facility audit and an index of process quality based on the observation of care delivered. We reviewed the WHO Service Availability and Readiness Assessment (SARA) to identify relevant structural quality indicators and the WHO Integrated Management of Childhood Illness (IMCI) for expected clinical actions and matched these to items available in the SPA survey. A total of 29 items on three domains were identified matching the SARA health facility readiness items (general readiness and readiness for the specific child curative care service) to assess the structure quality, using data extracted from the SPA health facility audits: (1) infrastructure (e.g., water, electricity, ambulance, etc.); (2) equipment, essential supplies and medications (e.g., gloves, sharps, medications storage, daily computer updates, etc.); (3) staffing and management (e.g., supervision provided, staff training, etc.). Moreover, 18 indicators were identified matching the IMCI items to assess the observed clinical care process quality. This process quality index covers assessment of clinical history, routine examination, and counseling on danger signs. To arrive at a facility-level process quality score, we first averaged structural indicators within facility, and then applied a rescaled patient and facility sampling weight to each clinical observation and averaged process indicators within observation, followed by averaging these clinical observations for sick child visits within each facility. We further averaged the facility-level structure and process quality score to arrive at a facility-level overall quality score to measure average performance on all indicators. For each quality index, indicators were averaged to provide a facility summary score from 0 to 1. Multiple imputation strategy was applied to address the missingness of individual indicators for facilities without sick child observations (up to 19%; see Appendix Table 1 for details), using the R Amelia package (33).

For peer review only - http://bmjopen.bmj.com/site/about/guidelines.xhtml

Sick child care utilization

Utilizing sick child care was defined as a binary indicator that children with diarrhea, fever, or symptoms of acute respiratory illness (ARI) sought curative care at formal health facilities. Following the MICS report in identifying which response options were considered as formal care (e.g. hospitals, health centers, clinics, health posts, and pharmacies), we did not include other sources of informal care (e.g. traditional healers or shops).

Covariates

We obtained data from the MES on household socioeconomic status (household wealth index, urban/rural residence), and demographics of caretaker and ill child (child's age and gender, mother's education level) as well as child illness type. The household wealth index was calculated following standard procedures for the DHS and classified into quintiles by the MES.

Patient and public involvement

Patients or the public were not involved in this study.

Statistical analysis

We obtained the spatial location of all enumeration areas (EAs) for the MICS from the 2008 Malawi census data. Each sick child was linked to the geographic centroid of the associated household's EA using EA codes provided by the Malawi National Statistical Office. SPA data include exact location of each facility. There was limited empirical evidence from Malawi regarding where children were taken to seek treatment for their illness. In this study, rather than assessing bypassing, we focused on whether having a high quality facility nearby promotes

For peer review only - http://bmjopen.bmj.com/site/about/guidelines.xhtml

For peer review only

household utilization of sick child health services. We matched each sick child and his/her household to the single nearest facility, based on travel distance by road to the EA centroid of the sick child's household. To calculate the geographic distance, we executed four steps: first identified the closet 8 facilities providing sick child care by using the geocoordinates for facilities (exact location) and household clusters (centroid); second, the road distance was calculated between cluster centroid and each facility based on the Google Maps API, including linear distance to nearest road where coordinates are not directly on a road (road type is not incorporated in this calculation); third, we replaced road distance with linear distance if road distance can not be calculated (e.g., if there is no road on an island) or if road distance is less than linear distance; lastly, we identified the facility with minimum distance to the cluster. Steps 1, 3, and 4 were executed in Stata, and Step 2 was run in Python 3.6.1. Based on prior studies suggesting that household distance to nearest health facility in Malawi is rarely greater than 50 km (34-36), we excluded children whose nearest facility was over 50 km away.

Descriptive analyses of facility quality for sick child care were first performed. We used logistic regression analyses of utilization of sick child care on the quality index and then adjusted for the covariates of interest. To understand which element of the overall quality served best as a predictor for household utilization of sick child care, we separated overall facility quality into structural and process quality.

We further conducted a series of sensitivity analyses to understand the robustness of our results. To understand whether the best performing facility may be more influential than the nearest facility, as long as it's still relatively accessible, we matched the household with the best performing facility within the buffer zone of a 5-km radius (direct distance from the household's EA centroid). Additionally, to test the sensitivity of catchment area definitions, we continued

For peer review only - http://bmjopen.bmj.com/site/about/guidelines.xhtml

using the best performing facility to match with the households, but modified the original 5-km buffer zones to 10-km and 20-km direct distance radius from the household's EA centroid.

Statistical analyses were run in Stata (version 14.1), mapping was done using QGIS Version 2.18 (Free Software Foundation, Massachusetts), and geographic distances (e.g., road traveling distances) were calculated based on Google Maps using Python 3.6.1.

RESULTS

Participants

In the 2013 SPA, 977 of a total of 1,066 surveyed health facilities (response rate: 92%) completed the assessment. Among these 977 facilities, 920 facilities (94%) offered sick child health services. Among these 920 facilities, 746 facilities had observations of sick child care, leaving 174 facilities that offer sick child services but did not have any observations of care. Completeness of each indicator is shown in Appendix Table 1. There was no missingness for infrastructure, but minimal for equipment, essential supplies and medications, substantial for supervision (up to 14%), and moderate for process quality (up to 19%).

The 2013 MICS dataset included 18,981 children under five years of age with completed caretaker interviews (response rate: 98%). Among these responding households, 52% of children (n=9,811) were reported by their caretakers as having symptoms of diarrhea (n=4,419, 45%), ARI (n=1,438, 15%), or fever (n=7,118, 73%). Among the 9,811 children who had illnesses, 6,679 children (68%) sought care at facilities (hospitals, health centers/clinics, or health posts/dispensaries).

In our main matching strategy, 110 of the 9,811 sick children were dropped due to the EAs for which locations are not available. Then, each of the 9701 sick children was matched

For peer review only - http://bmjopen.bmj.com/site/about/guidelines.xhtml

with the 920 health facilities to the child's single nearest facility based on road traveling distance to the EA centroid of the sick child's household. In this step, 352 health facilities were dropped. Therefore, our main matching strategy yielded an analytical sample of 568 health facilities providing sick child care services and 9,701 children who were reported by their caregivers as having illness of diarrhea, fever, or ARI. A total of 8,363 (86%) sick child consultations were actually observed in these selected facilities.

Descriptive Data

Table 1 detailed characteristics of health facilities in our main analytic sample. Among the health facilities that were included in the main analytic sample, health centers (57%) and clinics (26%) were the most common. The bulk of health facilities had clinical officers as the highest level of provider present (79%). The number of facilities located in rural areas was about four-fold those in urban settings, while about half were managed by government authority. Figure 1 shows the geographic distribution of all SPA health facilities and the ones included in our analytic sample, as well as the population density in the enumeration areas. Most facilities were located in the densely populated areas of Malawi.

Figure 1 to be inserted here.

For peer review only

For peer review only - http://bmjopen.bmj.com/site/about/guidelines.xhtml

Table 1. Facility characteristics and quality performance on sick child care services in the analytic sample (Main Model: n=568) [£]

	All facilities (568)	
	n or mean	% or SD
Rural[§]	445	78.4
Public[¶]	300	53.0
Facility type		
Hospital	75	13.2
Health center	323	56.9
Clinic	145	25.5
Health post	3	0.5
Other (pharmacy)	22	3.9
Highest clinician on site		
Medical doctor	60	10.6
Registered nurse	8	1.4
Enrolled nurse	38	6.7
Assistant medical officer	9	1.6
Clinical officer	448	78.9
Other health professional	5	0.9
Overall Quality Performance (mean, SD)		
Structural quality	0.62	0.14
Process quality	0.33	0.13
Overall quality	0.50	0.11

[£] n=568, the analytic sample was restricted to health service facilities, each of which was the single nearest to the household's EA centroid by road traveling distance. Facilities over 50 km away were excluded.
[§] Facility is in rural area.
[¶] Facility is managed by government authority.

For peer review only - http://bmjopen.bmj.com/site/about/guidelines.xhtml

Figures 2a and 2b detail the facility performance on a structural and process quality index, respectively. The average structural quality score for health facilities in Malawi providing sick child curative care services was 0.62 (SD: 0.14, range: 0.20- 0.97) and the average process quality score was 0.33 (SD: 0.14, range: 0.04- 0.78). The average overall quality score was 0.48 (SD: 0.10, range: 0.19- 0.90). Facilities were commonly equipped with basic infrastructure, such as client waiting rooms and general facility cleanliness, while they still lacked light sources, electricity, toilet, ambulance, and computer or internet access. Health facilities achieved high level performance on essential supplies and medication readiness, while about forty percent (40%) had sick child health service room infection control supplies. Within the staffing and management domain, nearly all facilities reported employing some form of supervision, while a modest number performed well in routine quality assurance or else received inadequate staff training on IMCI child health services. Reporting client opinions was extremely rare. With the exception of high percentages in process quality indicators achieved for fever, coughing, and temperature examination, all other indicators performed relatively modestly. The poorest indicator was providing counseling and examination for danger signs, edema, maternal-infant transmission of HIV, and ear pain.

Figure 2a to be inserted here.

Figure 2b to be inserted here.

Table 2 provides characteristics of sick children in the analytic sample, of whom, 6,679 children (69%) sought care at facilities during their illness, while 3,022 (31%) did not seek care. The average age of sick children was 29 months (SD: 16 months), with 16% younger than one year old. Forty-nine percent (49%) of the sick children were girls. The bulk of sick children had a perceived symptom of fever (64%), with ARI the least common (15%). The average road traveling distance to the nearest facility was about 5.8 km (SD: 4.7 km; median: 4.7 km).

For peer review only - http://bmjopen.bmj.com/site/about/guidelines.xhtml

329 **Table 2. Characteristics of sick child in the analytic sample (Main model: n=12,258)**

	**Total sick children (n=9,701)**		**Sought for care at facilities during illness (n= 6,679)**		**No care-seeking during illness (n= 3,022)**	
**Variable**	**n**	**%**	**n**	**%**	**n**	**%**
Child age (in months)						
≤ 2 months	162	1.7	88	1.3	74	2.5
2 months- 1 year	1,537	15.8	1,052	15.7	485	16.1
1 year - 5 years	8,002	82.5	5,539	82.9	2,463	81.5
Child sex						
Male	4,870	50.2	3,420	51.2	1,450	48.0
Female	4,831	49.8	3,259	48.8	1,572	52.0
Child sickness type						
Diarrhea	2,097	21.6	1,284	19.2	813	26.9
Fever	6,185	63.8	4,352	65.2	1,833	60.7
ARI	1,419	14.6	1,043	15.6	376	12.4
Mother's education						
None	1,287	13.2	817	12.2	470	15.6
Primary	7,016	72.3	4,828	72.3	2,188	72.4
Secondary or above	1,392	14.3	1,031	15.4	361	12.0
Household wealth quintile						
Q1 (poorest)	2,380	24.5	1,605	24.0	775	25.7
Q2	2,227	23.0	1,515	22.7	712	23.6
Q3	2,140	22.1	1,472	22.0	668	22.1
Q4	1,703	17.6	1,205	18.0	498	16.5

For peer review only - http://bmjopen.bmj.com/site/about/guidelines.xhtml

Q5 (richest)	1,251	13.0	882	13.2	369	12.2
Road Traveling Distance (km) to nearest facility (mean(SD), median)	5.77(4.7)	4.8	5.66(4.7)	4.70	6.00(4.7)	4.90

£ n=9,701, number of children who were perceived as sick in last two weeks by their caretakers that plausibly matched with their households' single nearest sick child health service facility in SPA. Facilities which are 50 km away from household's EA centroid were excluded.

Main findings

Table 3 show results of the multivariable logistic regression models of the association between quality of care and sick child care service utilization in Malawi health facilities. Model 1 and Model 4 represent the base and fully-specified models, respectively. Models 2 and 3 represent models adjusting for individual factors. Model 5 represents the fully specified model with the quality index separated into structural inputs and process quality.

The overall quality index of structural and process quality was a significant predicator of utilizing formal health facilities for sick child care services in Malawi. In the fully specified model (Model 4), the odds of utilizing formal health facilities increases by 56% (AOR: 1.56, SE: 0.31, 95% CI: 1.04, 2.28) for every hundred percent increase in the overall quality. The magnitude of the effect increases slightly when adjusting for the control variables, and remains significant in all models (Model 1-4). When separating the overall quality index into structural inputs and the process quality in the full specified model (Model 5), structural quality was a significant predictor (AOR: 1.42, SE: 0.25, 95% CI: 1.00, 2.01) while process quality was positively but not significantly associated with utilization (AOR: 1.29, SE: 0.23, 95% CI: 0.90, 1.83). The overall quality remained as a significant predicator for utilizing health facilities for sick child care in the three sensitivity analyses (SA) shown in Appendix Table 2. These SA

For peer review only

For peer review only - http://bmjopen.bmj.com/site/about/guidelines.xhtml

findings supported the association between facility quality and sick child care utilization even when using a large catchment area.

A child's type of illness and mother's education were found to be significant individual-level predictors of sick child care utilization. For children's illness type, in the fully specified model (Model 4) the results suggest higher odds of utilizing health facilities for children with a reported fever (AOR: 1.17, SE: 0.08, 95% CI: 1.02, 1.34) and symptoms of ARI (AOR:1.77, SE: 0.13, 95% CI: 1.53, 2.06), compared to those who were perceived to have diarrhea. This indicates that the severity of childhood illness is associated with motivating caregivers to utilize health facilities. For mother's education, the results suggest a gradient with increasing odds of utilizing health facilities as the level of the mother's education increases. Compared to mothers who did not have primary education, mothers who had primary education and those had secondary or higher education had 30% (AOR: 1.30, SE: 0.09, 95% CI: 1.13, 1.49) and 65% (AOR:1.65, SE: 0.15, 95% CI: 1.38, 1.97) higher odds of utilizing health facilities, respectively, after controlling for other factors.

For peer review only - http://bmjopen.bmj.com/site/about/guidelines.xhtml

365 **Table 3. Regression results for the association between sick child care utilization and the overall quality (structural and**
366 **process quality) of health service facility in Malawi.** £

**Main Models**	(1)		(2)		(3)		(4)		(5)	
VARIABLES	Odds ratio	SE	Adjusted odds ratio	SE	Adjusted odds ratio	SE	Adjusted odds ratio	SE	Adjusted odds ratio	SE
Overall quality	1.42*	(0.28)	1.47**	(0.29)	1.56**	(0.31)	1.56**	(0.31)	-	
Structural quality			-		-		-		1.42**	(0.25)
Process quality			-		-		-		1.29	(0.23)
Child age										
(Ref: ≤ 2 months)										
2 months- 1 year			1.99	(0.06)	1.00	(0.06)	1.00	(0.06)	1.04	(0.07)
1 year- 5 years			1.98***	(0.32)	1.99***	(0.32)	1.99***	(0.32)	1.99***	(0.35)
Child sex										
(Ref: Male)										
Female			1.14***	(0.05)	1.14***	(0.05)	1.14***	(0.05)	1.12**	(0.05)
Child sickness type										
(Ref: diarrhea)										
Fever			1.17**	(0.07)	1.17**	(0.08)	1.17**	(0.08)	1.21**	(0.09)

For peer review only - http://bmjopen.bmj.com/site/about/guidelines.xhtml

ARI			1.77***	(0.13)	1.78***	(0.13)	1.77***	(0.13)	1.79***	(0.16)
Mother education										
(Ref: No education)										
Primary					1.29***	(0.09)	1.30***	(0.09)	1.26***	(0.10)
Secondary or Higher					1.65***	(0.15)	1.65***	(0.15)	1.57***	(0.16)
Household Wealth Quintile (Ref: Q1 poorest)										
Q2					0.91	(0.07)	0.92	(0.08)	0.87	(0.08)
Q3					0.97	(0.08)	0.98	(0.09)	0.89	(0.09)
Q4					1.00	(0.08)	1.01	(0.09)	0.96	(0.09)
Q5					1.00	(0.08)	1.02	(0.09)	0.97	(0.09)
Household Rural Residence							0.96	(0.08)	0.92	(0.10)
Constant	0.38***	(0.04)	0.31***	(0.04)	0.24***	(0.03)	0.25***	(0.04)	0.424***	(0.04)
Observations	9,701		9,701		9,695		9,695		8,358	

standard error in parentheses

*** p<.01, ** p<.05, * p<.1

£ In the main models, the analytic sample was restricted to sick children taken by caregivers to seek care at their single nearest facility by road traveling distance. Facilities which are 50 km away were excluded.

For peer review only - http://bmjopen.bmj.com/site/about/guidelines.xhtml

For peer review only

DISCUSSION

In this paper, we sought to determine the influence of facility-based quality of care on care-seeking behaviors of Malawian parents faced with childhood illness. We found that facility quality is an important predictor of parental decision-making regarding care-seeking for illnesses such as diarrhea, fever, and ARI among children under five years, using national data from Malawi. Our sensitivity analyses varying the catchment areas consistently supported this association.

We found that about 69% of surveyed caregivers for sick children in our analytic sample utilized facility-based healthcare services in Malawi, reflecting a similar prevalence of care-seeking reported by the most recent two Malawi DHS surveys (7, 37). Although Malawi has been one of the top three countries with highest prevalence of care-seeking for children with reported fever, diarrhea, or symptoms of ARI in African maternal and child health (MCH) priority countries, care-seeking for sick under-five children was still not optimal given the target of ending preventable deaths of under five children by 2030 in Malawi Health Sector Strategic Plan (HSSP).

Our findings confirmed past research showing major gaps in service readiness and provider competence in sick child care. Like this work, others have noted poor provider performance, with fewer than half of clinical actions completed (e.g., taking patient history, examination, and counseling) (18, 20). Moreover, guideline adherence was low, with a high rate of missed diagnosis among children (four out of every five children) with pneumonia (38).

Our work advances on prior research in several ways. Past studies have largely focused on other factors in determining a caregiver's appropriate care-seeking for childhood illness, such as socio-demographic and household characteristics, social cultural factors, geographic access,

For peer review only - http://bmjopen.bmj.com/site/about/guidelines.xhtml

travel time, health facility fees, insurance, health worker densities, etc (39-43). Most prior studies on quality care for sick child care services were specific evaluations of quality improvement interventions, such as IMCI community health worker programs (44-46). Few studies have examined the relationship of quality and health care utilization on a national scale with regard to childhood illness in LMICs, with only one publication from Kenya attempting to construct the relationship between patient-perceived quality and the low attendance at the maternal and child health services using local district data.

When disaggregating our quality measure into structural and process components, we found that structural readiness (facility infrastructure, equipment, and staff) was a significant predictor of utilization, while process quality (adherence to medical guidelines) was positively but not significantly predictive of utilization. This finding suggests that individuals (caregivers) may weigh visible health facility characteristics (such as presence of client waiting room, general facility cleanness, equipment, drugs, etc.) more than they weigh the actual clinical care service experience. Because of the asymmetry of information between providers and patients in the healthcare market, patients do not have full knowledge about what constitutes good quality; in this case, they may not be fully aware of the recommended components of clinical assessment for their child (47).

This study had several strengths. First, the availability of exact spatial location data of the SPA health facilities and all EAs for the MICS from the 2008 Malawi census data provided a unique opportunity to examine health system and population data in concert to answer a policy relevant question. By contrast, DHS household locations are displaced up to 5km in rural areas, which precludes accurate matching to nearest facility. Second, we relied on WHO SARA and

For peer review only - http://bmjopen.bmj.com/site/about/guidelines.xhtml

IMCI guidelines to define quality measures. Furthermore, multiple sensitivity analyses confirmed that the findings were consistent across different facility matching specifications.

However, this study has several limitations. First, this study is based on cross-sectional data and is therefore unable to address causal claims. Second, given the available Malawi health system data (2013 MES), we chose the most clinically medical questions (variables) to indicate the demand by under-five children with sickness of diarrhea, malaria, or pneumonia. Our study focused on understanding the influence of quality on caregivers' decisions to utilize health facilities for their sick child. However, no patient outcome data were available, which prevented investigation of the linkage between facility quality and patient outcomes. Third, due to the data limitations, we extracted the quality index items available from the Malawi SPA dataset with reliance on the WHO SARA and IMCI, and therefore this analysis focused on specific health facility characteristics and interviewer-observed clinical quality service, which however do not assess individual perceptions of healthcare quality, nor address the patient-provider interpersonal quality of care. Moreover, in the real world, caretaker decisions in seeking care for a sick child, as well as where to seek care, can be influenced by their perception of the quality of child health services at facilities recently visited. Our study used the facility-based patient data that captures the nearest facility to the patient's household and we were not able to identify the previous facility which the patient visited. Given literature from other nations with comparable settings, in their first action, caregivers would seek care at the nearest facility upon recognizing the child's symptom; however, they would choose to bypass their nearest facility when it lacked diagnostic equipment, drugs, and skilled health workers, or had poor services (22, 48-50). In this study, we were not able to examine the influence of quality of the previous facility in relation to the caregiver's next health services facility choice, although these type of associations have been

For peer review only - http://bmjopen.bmj.com/site/about/guidelines.xhtml

beneficial in prior studies in developed countries (51). Thus, the matching strategy of linking a sick child to a health facility may not completely reflect actual behavior.

Policy Implications

Our findings have several implications for policy and future research. Our study provides strong and direct empirical evidence that better quality health facilities are associated with increased care utilization for childhood illness, which, if care is sufficiently competent, can improve odds of survival from treatable conditions. As our findings suggested, the government of Malawi may consider the improvement of the health facility structure, including equipment, essential supplies, drug storage and availability, room cleanness and infection control. Visible improvements are most likely to attract caregivers in utilization of the health facilities when they perceive childhood illness. However, beyond driving utilization, provider clinical competence needs to improve as well if visits are to be converted into better health. Given the low level of provider performance we and others have documented, this will require structure system reforms, potentially including updating pre-service education and re-organizing where care is delivered for maximum gains (6).

Future research is needed to validate and extend these findings in other country settings. Malawi has been a leader in sub-Saharan Africa, demonstrating strong political will to implement evidence-based interventions that can improve maternal and child health. The availability of the geocoded health facilities and population data enabled us to match the health system facility survey data and the population data in. Other countries could take similar measures to permit matching of health system and household data to obtain better insights in how health systems influence health and care seeking. These measures would aid in policy

For peer review only - http://bmjopen.bmj.com/site/about/guidelines.xhtml

462 determinations, to evaluate whether services supplied by the health system can actually meet
463 with the demands of the population, and quality of care can satisfy the population's needs and
464 promote patient care-seeking behaviors that reduce preventable deaths. In addition, the facility
465 quality index needs to be validated in different country settings, encompassing higher mortality
466 burdens and different health system capacities, to strengthen the generalizability of the results.

467 As an important component of human capital, health can contribute positively to a
468 nation's economic development (52). At the same time, achieving an excellent state of health is
469 an intrinsic part of the goals of social development, as well as an essential factor in an
470 individual's well-being. Good quality health care is thus both an intrinsic good that can promote
471 health outcomes as well as a driver of utilization; to achieve these ends both structures and
472 processes of care need to improve.

For peer review only

For peer review only - http://bmjopen.bmj.com/site/about/guidelines.xhtml

Acknowledgements

Earlier versions of this paper were presented during several seminars at the Harvard Global Health Institute, the Harvard T.H. Chan School of Public Health, and the Fifth Global Symposium on Health Systems Research, where valuable comments were received from seminar participants, including Stéphane Verguet, Michael Reich and Sara Singer. The authors thank Dennis Lee and Fei Carnes for assistance with geographic linking. The authors also acknowledge Humphreys Nsona for his input on the initial study idea and assistance with the geospatial data.

Contributors: MEK conceptualized the study. HHL and LL curated data. LL and HHL conducted the formal analysis and designed the methodology. LL visualized the data results and wrote the original draft. All authors reviewed and contributed to the editing of the manuscript. All authors approved the final manuscript submitted.

Funding: This research received no specific grant from any funding agency in the public, commercial or not-for-profit sectors.

Competing Interests Statement: None declared.

Patient Consent: Not required.

Ethical approval: The Harvard University Human Research Protection Program deemed this analysis of secondary data exempt from human subject review.

Data Sharing Statement: SPA data files are available from the DHS Program (http://dhsprogram.com/whatwedo/survey/survey-display-424.cfm). MICS survey are available from UNICEF (http://mics.unicef.org/surveys); cluster sampling details were provided on request from the Malawi National Statistical Office.

For peer review only - http://bmjopen.bmj.com/site/about/guidelines.xhtml

References

1. The United Nations Millennium Development Goals. [cited 2017 September 20]. Available from: http://www.un.org/millenniumgoals/.
2. The United Nations Sustainable Development Goals. Available from: http://www.un.org/sustainabledevelopment/sustainable-development-goals/.
3. World Health Organization. World Health Report 2013: Research for Universal Health Coverage. 2013.
4. United Nations General Assembly. GA/11326. [cited 2017 October 20]. Available from: http://www.un.org/en/ga/67/resolutions.shtml.
5. Berwick DM, Kelley E, Kruk ME, Nishtar S, Pate MA. Three global health-care quality reports in 2018. The Lancet. 2018;392(10143):194-5.
6. Kruk ME, Gage AD, Arsenault C, Jordan K, Leslie HH, Roder-DeWan S, et al. High-quality health systems in the Sustainable Development Goals era: time for a revolution. The Lancet Global Health. 2018;6(11):e1196-e252.
7. National Statistical Office (NSO) [Malawi] and ICF. Malawi Demographic and Health Survey 2015-16. Zomba, Malawi, and Rockville, Maryland, USA. NSO and ICF: 2017.
8. Central Statistical Agency (CSA) [Ethiopia] and ICF. Ethiopia Demographic and Health Survey 2016. Addis Ababa, Ethiopia, and Rockville, Maryland, USA: CSA and ICF: 2016.
9. Ministry of Health CD, Gender, Elderly and Children (MoHCDGEC) [Tanzania Mainland], Ministry of Health (MoH) [Zanzibar], National Bureau of Statistics (NBS), Office of the Chief Government Statistician (OCGS), and ICF. Tanzania Demographic and Health Survey and Malaria Indicator Survey (TDHS-MIS) 2015-16. Dar es Salaam, Tanzania, and Rockville, Maryland, USA: MoHCDGEC, MoH, NBS, OCGS, and ICF: 2016.
10. Kenya National Bureau of Statistics MoHK, National AIDS Control Council/Kenya, Kenya Medical Research Institute, National Council for Population and Development/Kenya, and ICF International. Kenya Demographic and Health Survey 2014. Rockville, MD, USA: Kenya National Bureau of Statistics, Ministry of Health/Kenya, National AIDS Control Council/Kenya, Kenya Medical Research Institute, National Council for Population and Development/Kenya, and ICF International: 2015.
11. United Nations Development Porgramme (UNDP). 2014 Millennium Development Goal Report for Malawi. United Nations Development Programme, New York: 2014.
12. The Demographic and Health Surveys. Malawi Service Provision Assessment (SPA) 2013-14.
13. National Statistical Office/Malawi and Macro International. Malawi Demographic and Health Survey 1992. Zomba, Malawi: National Statistical Office/Malawi and Macro International: 1994.
14. Sobel HL, Huntington D, Temmerman M. Quality at the Centre of Universal Health Coverage. Health policy and planning. 2015;31(4):547-9.
15. Hanefeld J, Powell-Jackson T, Balabanova D. Understanding and Measuring Quality of Care: Dealing with Complexity. Bulletin of the World Health Organization. 2017;95(5):368.
16. Kruk ME, Larson E, Twum-Danso NA. Time for a Quality Revolution in Global Health. The Lancet Global health. 2016;4(9):e594-e6.
17. Abera Abaerei A, Ncayiyana J, Levin J. Health-care Utilization and Associated Factors in Gauteng Province, South Africa. Global Health Action. 2017;10(1):1305765.
18. Leslie HH, Ndiaye Y, Kruk ME. Effective coverage of primary care services in eight high-mortality countries. BMJ Global Health. 2017;2(3):e000424.

For peer review only - http://bmjopen.bmj.com/site/about/guidelines.xhtml

19. Gera T, Shah D, Garner P, Sachdev HS. Integrated Management of Childhood Illness (IMCI) Strategy for children under five: effects on death, service utilisation and illness. Cochrane Database of Systematic Reviews. 2012(9).
20. Kruk ME, Gage AD, Mbaruku GM, Leslie HH. Content of Care in 15,000 Sick Child Consultations in Nine Lower-Income Countries. Health services research. 2018.
21. Uwemedimo OT, Lewis TP, Essien EA, Chan GJ, Nsona H, Kruk ME, et al. Distribution and determinants of pneumonia diagnosis using Integrated Management of Childhood Illness guidelines: a nationally representative study in Malawi. BMJ global health. 2018;3(2):e000506.
22. Audo M, Ferguson A, Njoroge P. Quality of Health Care and Its Effects in the Utilisation of Maternal and Child Health Services in Kenya. East African Medical Journal. 2005;82(11).
23. Creanga AA, Gullo S, Kuhlmann AKS, Msiska TW, Galavotti C. Is quality of care a key predictor of perinatal health care utilization and patient satisfaction in Malawi? BMC pregnancy and childbirth. 2017;17(1):150.
24. Kruk ME, Mbaruku G, McCord CW, Moran M, Rockers PC, Galea S. Bypassing primary care facilities for childbirth: a population-based study in rural Tanzania. Health policy and planning. 2009;24(4):279-88.
25. Gage AD, Leslie HH, Bitton A, Jerome JG, Thermidor R, Joseph JP, et al. Assessing the quality of primary care in Haiti. Bulletin of the World Health Organization. 2017;95(3):182.
26. World Bank Data Indicators [Internet]. [cited March 03, 2017]. Available from: http://data.worldbank.org/.
27. National Statistical Office. Malawi MDG Endline Survey 2014. Zomba, Malawi: National Statistical Office; 2015
28. UNICEF, WHO, World Bank, UN-DESA Population Division. Levels and Trends in Child Mortality Report 2017. New York: United Nations Children's Fund, 2017.
29. Kruk ME, Pate M, Mullan Z. Introducing The Lancet Global Health Commission on High-quality Health Systems in the SDG Era. The Lancet Global Health. 2017;5(5):e480-e1.
30. The Institute of Medicine. Crossing The Quality Chasm: A New Health System For The 21st Century. 2001
31. Donabedian A. Evaluating the Quality of Medical Care. Milbank Quarterly. 2005;83(4):691-729.
32. Campbell SM, Roland MO, Buetow SA. Defining Quality of Care. Social Science & Medicine. 2000;51(11):1611-25.
33. Honaker J, King G, Blackwell M. Amelia II: A program for missing data. Journal of statistical software. 2011;45(7):1-47.
34. Ewing VL, Lalloo DG, Phiri KS, Roca-Feltrer A, Mangham LJ, SanJoaquin MA. Seasonal and geographic differences in treatment-seeking and household cost of febrile illness among children in Malawi. Malar J. 2011;10(1):32.
35. Karra M, Fink G, Canning D. Facility distance and child mortality: a multi-country study of health facility access, service utilization, and child health outcomes. Int J Epidemiol. 2016;46(3):817-26.
36. Malawi National Statistical Office. Malawi Demographic and Health Survey 1992 National Statistical Office, Zomba, Malawi, 1992.
37. National Statistical Office (NSO) and ICF Macro. Malawi Demographic and Health Survey 2010. Zomba, Malawi, and Calverton, Maryland, USA: NSO and ICF Macro: 2011.
38. Omolara T. Uwemedimo, Todd P. Lewis, Elsie A. Essien, Grace J. Chan, Humphreys Nsona, Margaret E. Kruk, et al. Distribution and determinants of pneumonia diagnosis using

For peer review only - http://bmjopen.bmj.com/site/about/guidelines.xhtml

Integrated Management of Childhood Illness guidelines: a nationally representative study in Malawi. BMJ Global Health. Forthcoming.
39. Abdulkadir MB, Abdulkadir ZA. A Cross-sectional Survey of Parental Care-seeking Behavior for Febrile Illness Among Under-five Children in Nigeria. Alexandria Journal of Medicine. 2017;53(1):85-91.
40. Noordam AC, Carvajal-Velez L, Sharkey AB, Young M, Cals JW. Correction: Care Seeking Behaviour for Children with Suspected Pneumonia in Countries in Sub-Saharan Africa with High Pneumonia Mortality. PloS one. 2015;10(4):e0126997.
41. Shaw B, Amouzou A, Miller NP, Bryce J, Surkan PJ. A Qualitative Exploration of Care-seeking Pathways for Sick Children in the Rural Oromia Region of Ethiopia. BMC Health Services Research. 2017;17(1):184.
42. Chibwana AI, Mathanga DP, Chinkhumba J, Campbell CH. Socio-cultural Predictors of Health-seeking Behaviour for Febrile Under-five Children in Mwanza-Neno District, Malawi. Malaria Journal. 2009;8(1):219.
43. Hjortsberg C. Why Do the Sick Not Utilise Health Care? The Case of Zambia. Health Economics. 2003;12(9):755-70.
44. Cardemil CV, Gilroy KE, Callaghan-Koru JA, Nsona H, Bryce J. Comparison of Methods for Assessing Quality of Care for Community Case Management of Sick Children: an Application With Community Health Workers in Malawi. The American Journal of Tropical Medicine and Hygiene. 2012;87(5_Suppl):127-36.
45. Miller NP, Amouzou A, Tafesse M, Hazel E, Legesse H, Degefie T, et al. Integrated Community Case Management of Childhood Illness in Ethiopia: Implementation Strength and Quality of Care. The American Journal of Tropical Medicine and Hygiene. 2014;91(2):424-34.
46. Nsona H, Mtimuni A, Daelmans B, Callaghan-Koru JA, Gilroy K, Mgalula L, et al. Scaling Up Integrated Community Case Management of Childhood Illness: Update from Malawi. The American Journal of Tropical Medicine and Hygiene. 2012;87(5_Suppl):54-60.
47. Arneill AB, Devlin AS. Perceived Quality of Care: The Influence of the Waiting Room Environment. Journal of Environmental Psychology. 2002;22(4):345-60.
48. Kruk ME, Hermosilla S, Larson E, Mbaruku GM. Bypassing Primary Care Clinics for Childbirth: a Cross-sectional Study in the Pwani Region, United Republic of Tanzania. Bulletin of the World Health Organization. 2014;92(4):246-53.
49. Kahabuka C, Kvåle G, Moland KM, Hinderaker SG. Why Caretakers Bypass Primary Health Care Facilities for Child Care-a Case From Rural Tanzania. BMC health services research. 2011;11(1):315.
50. Lohela TJ, Campbell OM, Gabrysch S. Distance to Care, Facility Delivery and Early Neonatal Mortality in Malawi and Zambia. PloS one. 2012;7(12):e52110.
51. Jung K, Feldman R, Scanlon D. Where Would You Go For Your Next Hospitalization? Journal of Health Economics. 2011;30(4):832-41.
52. Bloom DE, Canning D, Sevilla J. The Effect of Health on Economic Growth: a Production Function Approach. World Development. 2004;32(1):1-13.

For peer review only

For peer review only - http://bmjopen.bmj.com/site/about/guidelines.xhtml

Figure 1. Distribution of health facilities in Malawi relative to MES enumeration areas. £

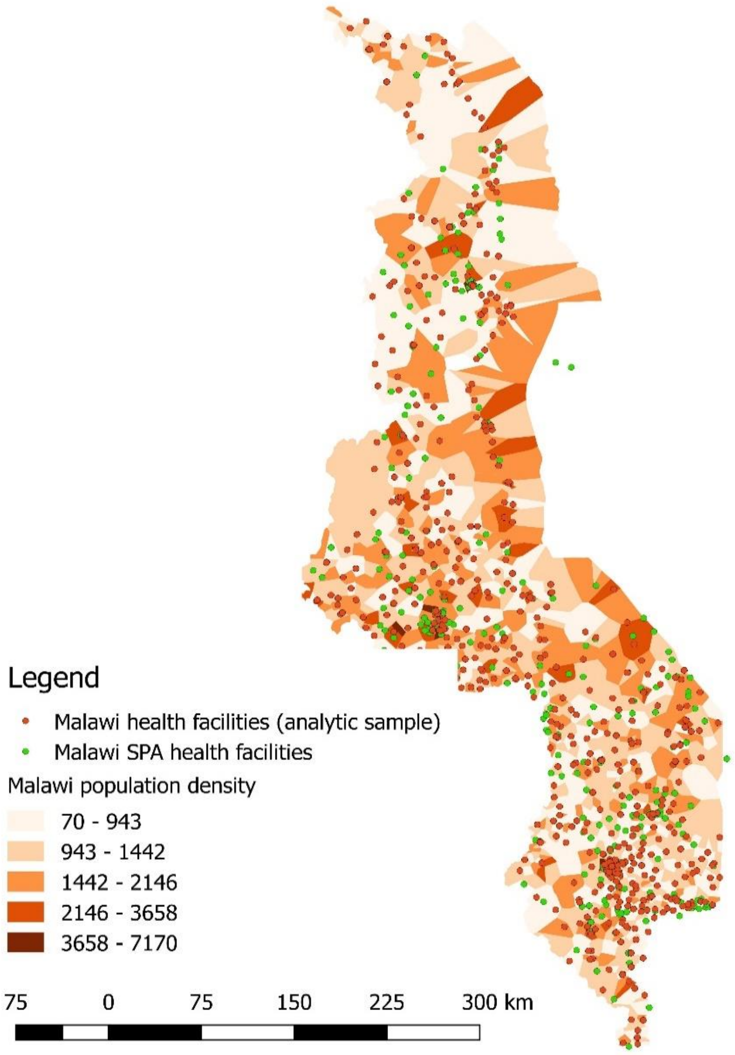

£ This map reflects the distribution of all SPA health facilities and the health facilities included in the analytic sample, as well as the population density in Malawi.

Figure 2a. Performance on facility structural quality index: percentage of facilities with key resources and services (n=568).

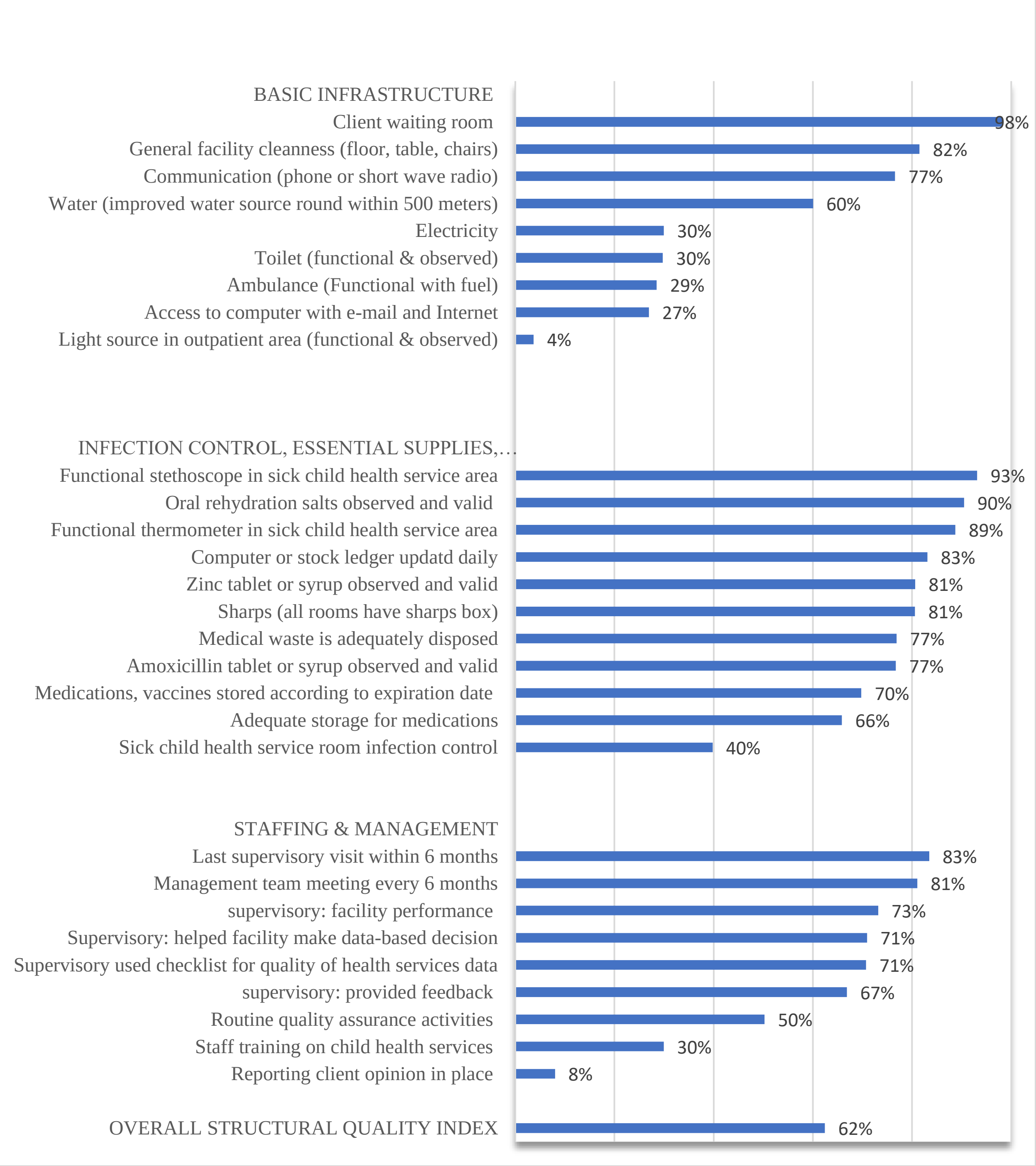

£ n=568, the analytic sample was restricted to health service facilities, each of which was the single nearest to the household's EA centroid by road traveling distance. Facilities over 50 km away were excluded.

Figure 2b. Performance on facility process quality index: percentage of facilities with key resources and services (n=568).

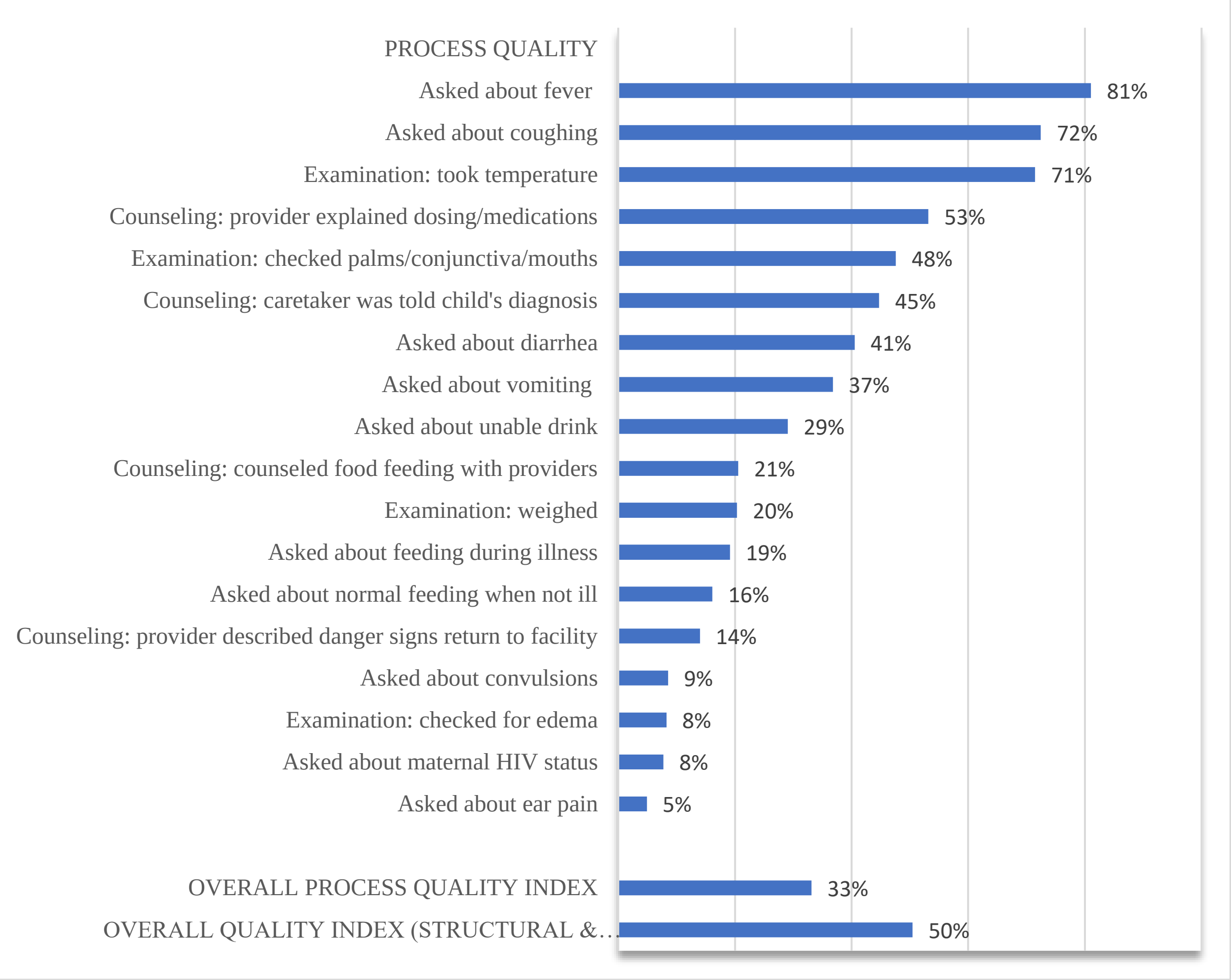

[£] n=568, the analytic sample was restricted to health service facilities, each of which was the single nearest to the household's EA centroid by road traveling distance. Facilities over 50 km away were excluded.

Appendix Table 1: Completeness of indicators for facilities† (n=920) and observations

Variable	% Missing	% Completed
Water (improved water source round within 500 meters)	0	100
Ambulance (Functional with fuel)	0	100
Electricity	0	100
Communication (phone or short wave radio)	0	100
Toilet (functional & observed)	0	100
General facility cleanness (floor, table, chairs)	0	100
Client waiting room	0	100
Access to computer with e-mail and Internet	0	100
Light source (functional & observed)	0	100
Sick child health service room infection control	0	100
Sharps (all rooms have sharps box)	0.33	99.67
Medical waste is adequately disposed	0.43	99.57
Functional thermometer in sick child health service area	0	100
Functional stethoscope in sick child health service area	0	100
Oral rehydration salts in pharmacy or sick child health service area	0	100
Amoxicillin tablet or syrup observed and valid	0.65	99.35
Zinc tablet or syrup observed and valid	0.65	99.35
Medications, vaccines stored according to expiration date	11.96	88.04
Adequate storage for medications	0	100
Computer or stock ledger updated daily	0	100
Staff training on child health services	0	100
Last supervisory visit within 6 months	0	100
Management team meeting every 6 months	0.65	99.35
Supervisory used checklist for quality of health services data	13.70	86.30

For peer review only - http://bmjopen.bmj.com/site/about/guidelines.xhtml

Supervisory: facility performance	13.70	86.30
Supervisory: helped facility make data-based decision	13.70	86.30
Supervisory: provided feedback	13.70	86.30
Reporting client opinion in place	1.20	98.8
Routine quality assurance activities	0.87	99.13
Asked about vomiting	18.91	81.09
Asked about unable to drink	18.91	81.09
Asked about convulsions	18.91	81.09
Asked about normal feeding when not ill	18.91	81.09
Asked about maternal HIV status	18.91	81.09
Asked about feeding during illness	18.91	81.09
Asked about fever	18.91	81.09
Asked about ear pain	18.91	81.09
Asked about diarrhea	18.91	81.09
Asked about coughing	18.91	81.09
Examination: weighed	18.91	81.09
Examination: took temperature	18.91	81.09
Examination: checked palms/conjunctiva/mouths	18.91	81.09
Examination: checked for edema	18.91	81.09
Counseling: provider explained dosing/medications	19.24	80.76
Counseling: counseled food feeding with providers	18.91	81.09
Counseling: caretaker was told child's diagnosis	18.91	81.09
Counseling: provider described danger signs return to facility	18.91	81.09

† A total of 920 health facilities from 2013 Malawi SPA that provided child curative care was included in this study.

For peer review only - http://bmjopen.bmj.com/site/about/guidelines.xhtml

Appendix Table 2. Sensitivity analyses model results for the association between sick child care utilization and the overall quality (structural and process quality) of health service facility in Malawi.

**Model**	**Adjusted results** †		**N**
	Odds Ratio		
Main Model: the single nearest facility	1.56**	(0.31)	9,695
Sensitivity Analysis 1: the best facility within 5 km	2.49***	(0.60)	6,429
Sensitivity Analysis 2: the best facility within 10 km	1.95***	(0.38)	9,296
Sensitivity Analysis 3: the best facility within 20 km	2.20***	(0.46)	9,682

standard error in parentheses

*** p<.01, ** p<.05, * p<.1

†Adjusted for: sick child age, sex, type of illness, mother education, household wealth quintile, and the household's rural residence.

For peer review only - http://bmjopen.bmj.com/site/about/guidelines.xhtml

BMJ Open

Exploring the association between sick child healthcare utilization and health facility quality in Malawi: A Cross-Sectional Study

Journal:	*BMJ Open*
Manuscript ID	bmjopen-2019-029631.R1
Article Type:	Research
Date Submitted by the Author:	08-May-2019

Complete List of Authors:	LIU, LINGRUI; Yale University School of Public Health, Health Policy and Management; Global Health Leadership Initiative Yale University<br>Leslie, Hannah; Harvard TH Chan School of Public Health, Department of Global Health and Population<br>Joshua, Martias; Malawi Ministry of Health<br>Kruk, Margaret; Harvard T.H. Chan School of Public Health , Department of Global Health and Population

**Primary Subject Heading**:	Global health
Secondary Subject Heading:	Health services research
Keywords:	health facility quality, sick child healthcare, healthcare utilization, Malawi, sub-Saharan Africa

SCHOLARONE™ Manuscripts

Title

Exploring the association between sick child healthcare utilization and health facility quality in Malawi: A Cross-Sectional Study

Authors

Lingrui Liu[1,2], Hannah H. Leslie[3], Martias Joshua[4], Margaret E. Kruk[3]

Authors' Affiliations

[1]Department of Health Policy and Management, Yale School of Public Health, New Haven, CT, USA
[2]Global Health Leadership Initiative, Yale University, New Haven, CT, USA
[3]Department of Global Health and Population, Harvard T.H. Chan School of Public Health, Boston, MA, USA
[4]Malawi Ministry of Health, Lilongwe, Malawi

Corresponding author:

Lingrui Liu
Department of Health Policy and Management
Yale School of Public Health
New Haven, Connecticut 06510, USA
Lingrui.liu@yale.edu

Word Count (excluding title page, abstract, references, figures and tables) : 4983

For peer review only

For peer review only - http://bmjopen.bmj.com/site/about/guidelines.xhtml

ABSTRACT (word count: 299)

Objective: Increasing the availability of basic healthcare services in low-and-middle-income countries is not sufficient to meet the Sustainable Development Goal target for child survival in high-mortality settings, where healthcare utilization is often inconsistent and quality of care can be poor. We assessed whether poor quality of sick child healthcare in Malawi is associated with low utilization of sick child healthcare.

Design: We measured two elements of quality of sick child healthcare: facility structural readiness and process of care using data from the 2013 Malawi Service Provision Assessment. Overall quality was defined as the average of these metrics. We extracted demographic data from the 2013/2014 Malawi Multiple Indicator Cluster Survey and linked households to nearby facilities using geocodes. We used logistic regression to examine the association of facility quality with utilization of formal health services for children under five suffering diarrhea, fever, or cough/acute respiratory illness, controlling for demographic and socioeconomic characteristics. We conducted sensitivity analyses, modifying the travel distance and population - facility matching criteria.

Setting and Population: 568 facilities were linked with 9,701 children with recent illness symptoms in Malawi, of whom 69% had been brought to a health facility.

Results: Overall, facilities showed gaps in structural quality (62% readiness) and major deficiencies in process quality (33%), for an overall quality score of 48%. Better facility quality was associated with higher odds of utilization of sick child healthcare services (Adjusted odds ratio [AOR]: 1.66, 95% CI:1.04, 2.63), as was structural quality alone (AOR: 1.33, 95% CI:0.95, 1.87). Sensitivity analyses supported the main finding.

Conclusion: Although Malawi's health facilities for curative child care are widely available, quality and utilization of sick child healthcare services are in short supply. Improving facility

For peer review only - http://bmjopen.bmj.com/site/about/guidelines.xhtml

quality may provide a way to encourage higher utilization of healthcare and thereby decrease preventable childhood morbidity and mortality.

Keywords: health facility quality, sick child healthcare, healthcare utilization, Malawi, sub-Saharan Africa

Strengths and limitations of this study

- Using the spatial geocodes, this study linked the health system (Malawi SPA) and the household (2013 Malawi MICS) datasets to investigate the role of quality in sick child healthcare utilization in Malawi.
- This study relied on internationally endorsed guidelines to define quantifiable quality of care measures.
- Multiple sensitivity analyses confirmed the findings.
- However, matching strategy of linking a sick child to a health facility may not completely reflect actual behavior.
- We acknowledged that the MICS survey data on symptoms of illness do not provide sufficient specificity on illness severity and thus were unable to determine that all children with these symptoms in fact require formal healthcare at health facilities.

For peer review only

For peer review only - http://bmjopen.bmj.com/site/about/guidelines.xhtml

INTRODUCTION

The global health community has achieved notable gains in the Millennium Development Goals (MDGs) era. However, elimination of preventable and treatable child mortality remains an urgent global health priority in the coming decade (1, 2). Over the past decade under the umbrella goal of universal health coverage (UHC) (3, 4), health policies have focused on the expansion of coverage of essential health interventions and basic services in sub-Saharan Africa (SSA) and other low-income regions. Due to the rising recognition that, without improving quality of care in the health system, improved access to healthcare alone would not achieve expected health outcomes, the global health community has begun to focus on improvement of healthcare quality (5, 6). Large expansions of health facility networks have been attained in SSA and other low-income regions, and yet, utilization of available resources for care of sick children under five remains low, resulting in inadequate care-seeking for children with diarrhea, malaria or pneumonia (7-10). Malawi, a sub-Saharan African country whose government has declared reduction of preventable children-under-five mortality as a national priority, achieved the MDG targets for improved child health (11). However, healthcare utilization for conditions treatable by the health system is low, as data from the most recent Demographic and Health Survey (DHS) 2015 in Malawi shows (12). Although healthcare facility utilization increased steadily from 2000 to 2015, the proportion of sick children under five with symptoms of acute respiratory infection (ARI), fever, and diarrhea who were taken to a health provider for treatment within 48 hours of symptom onset, remained insufficient (51%, 46%, and 66%, respectively) (7). These utilization patterns occur despite wide availability of child health services (including outpatient curative care, child growth monitoring, and child vaccination) in Malawi's health facilities (12), with a median distance to the nearest health facility of 5 km and an estimated median traveling time of

For peer review only - http://bmjopen.bmj.com/site/about/guidelines.xhtml

75 minutes (13). There is a growing recognition that, besides geographic access, poor quality of care could be a supply-side barrier to UHC, deterring patients from obtaining treatment and influencing family decisions to use or avoid services, which would ultimately impact health outcome gains (14-17).

Although Malawi has achieved substantial improvement in coverage (utilization by those in need) for curative care in children, as estimated from household survey data, quality remains weak (18, 19). Moreover, a recent multi-country study demonstrated that the duration and the content of sick child healthcare service was inadequate (20). Another recent study echoed that care quality in pneumonia diagnosis for a sick child in Malawi is poor, with low guideline adherence to the Integrated Management of Childhood Illness (IMCI) criteria (21). Most existing evaluation studies on the quality of sick child healthcare delivered at health facilities in Malawi have aimed to describe the state of quality but few have tackled the question of whether poor quality dissuades families from using the health system when a child falls ill. In the area of reproductive health, one study suggested a positive association between the perceived quality of reproductive care at the facility last attended and the family utilization of immunization and treatment services for their children at these health facilities, as observed within facilities in Kisumu Municipality in Western Kenya (22). Another recent research from a health intervention program in Ntcheu district in Malawi identified that quality of perinatal care provided is an important predictor of both women's use and satisfaction with such services received (23). Furthermore, earlier literature on bypassing for facility delivery in rural Africa has documented that quality of care is influencing clinic choice (24). Although these studies suggested a positive association between respondent perception of quality and utilization, it would be worthwhile to investigate this relationship at a broader population level (e.g., using national household-

For peer review only - http://bmjopen.bmj.com/site/about/guidelines.xhtml

representative data), as well as through use of the most recent health facility assessment standardized surveys, to explore the relationship.

Policymakers need evidence on what health system attributes (e.g., quality of sick child care) may influence primary care utilization. Malawi is focusing on health sector strategies to improve child health and wellbeing, and has embarked on its second Health Sector Strategic Plan (HSSP 2011-2016), making this an opportune time to seek evidence on the health system factors (e.g., quality) that best promote utilization of sick child healthcare services. In this paper we examine the association between quality of care in health facilities in Malawi and utilization of sick child healthcare by caregivers. This study is one of the few which we are aware that focuses on the contribution of facility quality to utilization of child healthcare services in high-disease-burden settings (25).

METHODS

Study Sample

Malawi is one of the smallest and most densely-populated countries in sub-Saharan Africa, made up of a predominantly young population, of which almost half is aged 15 years or younger, and more than 20% children under five. With a total population of 17 million and a gross domestic product (GDP) per capita of US$1200 in 2015, approximately 52% of Malawi's population lives below the international poverty line ($1.90 per person per day in 2011 purchasing power parity [PPP] dollars), with more than 80% inhabiting rural regions (26). Health facilities that provide child health services in the formal sector of Malawi include hospitals, health centers, clinics, dispensaries, and health posts.

For peer review only - http://bmjopen.bmj.com/site/about/guidelines.xhtml

The analysis in this research combined data from multiple sources. To obtain information on the facility quality of child health services, we used the Malawi 2013-14 Service Provision Assessment (SPA), a comprehensive census of all formal-sector health facilities conducted by the Demographic and Health Surveys (DHS) Program (12). SPA is a standardized cross-sectional survey of public and private health facilities, which includes a facility-level audit, clinical observation protocols, patient exit interviews, and healthcare provider interviews. The health facility audit was conducted in each health facility visited using a standardized inventory questionnaire. At least one health worker was interviewed in a sampled health facility. Interviewers also observed a sample of patient-provider clinical care service interaction processes, as well as interviewing patients observed receiving care upon their exit from the health facility. The SPA survey does not report or examine clinical outcomes. In this study, we examined the quality of out-patient sick child curative care delivered at health facilities in Malawi, excluding child vaccination services and child growth monitoring services. We limited our sample of facilities to those facilities (including hospitals, health centers, health posts, dispensaries) which provided outpatient sick child curative care services.

To obtain household information and care-seeking for children under five years of age, we used the 2013/2014 Malawi Multiple Indicator Cluster (27). Malawi 2013 MICS is a household survey conducted to assess health outcomes among a nationally-representative sample of the population, employing a multi-stage sampling strategy. Enumeration areas (EAs) were sampled within the strata of district and urban versus rural location, and then households were identified within EAs. With a systematic sample of 25 households drawn in each sample cluster, a total sample of 1140 EAs and 28,479 households were selected for the Malawi 2013 MICS. We limited our sample of households to those in which caregivers reported children under five years

For peer review only - http://bmjopen.bmj.com/site/about/guidelines.xhtml

of age who had an episode of diarrhea, symptoms of acute respiratory infection (ARI), or fever during the two weeks prior to the survey. Although these three specific medical conditions cannot cover the full range of illnesses of children under five, malaria, diarrhea, and pneumonia remain leading causes of death among children under age five and are the most consistent indicators for child disease burden across the globe, with the vast majority occurring in LMICs (28). These conditions combined are relevant in epidemiological predictions and represent demand for sick child healthcare services in Malawi.

Measure of quality of child health services delivered at health facilities in Malawi

To date, there has not been a single uniform set of measures on quality definition and metrics (15, 29). The Institute of Medicine report *Crossing the Quality Chasm* identified six dimensions to measure quality: safe, effective, patient-centered, timely, efficient, and equitable (30). We consolidated these dimensions with Donabedian's (1980) quality of care framework of structure, process and outcome (31). The structure elements describe the characteristics of the environment in which healthcare is provided, which exist before the care takes place. The structural inputs of a health system indicate the capability and serviceability of a health system under which care occurs (32). Process features include two key components: technical interventions (appropriate delivery of clinical procedures following clinical guidelines, e.g., WHO Integrated Management of Childhood Illness [IMCI] guidelines in child curative care services) and inter-personal interactions between users and a healthcare system. Outcomes refer to the final consequences of healthcare, such as the under-five mortality rate (32). In this study, available data did not specify patient outcomes for the children experiencing recent illness.

For peer review only - http://bmjopen.bmj.com/site/about/guidelines.xhtml

Using Donabedian's framework, we constructed an index of structural quality based on the facility audit and an index of process quality based on the observation of healthcare delivered. We reviewed the WHO Service Availability and Readiness Assessment (SARA) to identify relevant structural quality indicators and the WHO Integrated Management of Childhood Illness (IMCI) for expected clinical actions and matched these to items available in the SPA survey. A total of 29 items on three domains were identified matching the SARA health facility readiness items (general readiness and readiness for the specific child curative care service) to assess the structure quality, using data extracted from the SPA health facility audits: (1) infrastructure (e.g., water, electricity, ambulance, etc.); (2) equipment, essential supplies and medications (e.g., gloves, sharps, medications storage, daily computer updates, etc.); (3) staffing and management (e.g., supervision provided, staff training, etc.). Moreover, 18 indicators were identified matching the IMCI items to assess the observed clinical care process quality. This process quality index covers assessment of clinical history, routine examination, and counseling on danger signs. We first averaged structural indicators within facility. We then averaged process indicators within observation. Further, we averaged these clinical observations for sick child visits within each facility to obtain the facility level process quality score, using a rescaled weight for each clinical observation to reflect the sampling probability of patients within facilities. We further averaged the facility-level structure and process quality score to arrive at a facility-level overall quality score to measure average performance on all indicators. For each quality index, indicators were averaged to provide a facility summary score from 0 to 1. Multiple imputation was applied to address the missingness of individual indicators for facilities without sick child observations (up to 19%; see Appendix Table 1 for details), using the R Amelia package (33). Considering the multiple imputation assumption that missingness is random conditional on the covariates, we

For peer review only - http://bmjopen.bmj.com/site/about/guidelines.xhtml

included four covariates in our imputation: facility type (e.g., central hospital, district hospital, health centers, etc.), facility managing authority (e.g., government, private, NGO, mission/faith-based, etc.), districts, and urban/rural.

Sick child care utilization

Utilizing sick child healthcare was defined as a binary indicator that children with diarrhea, fever, or symptoms of acute respiratory illness (ARI) sought curative care at formal health facilities. Following the MICS report in identifying which response options were considered as formal care and being consistent with the facility types surveyed in SPA, we consider health facilities including hospitals, health centers, clinics, health posts, and dispensaries, and did not include other sources of informal care (e.g. traditional healers or shops).

Covariates

We obtained data from the MICS on household socioeconomic status (household wealth index, urban/rural residence), and demographics of caretaker and ill child (child's age and gender, mother's education level) as well as child illness type. The household wealth index was calculated following standard procedures for the DHS and classified into quintiles by the MICS.

Patient and public involvement

Patients or the public were not involved in this study.

Statistical analysis

We obtained the spatial location of all enumeration areas (EAs) for the MICS from the

For peer review only - http://bmjopen.bmj.com/site/about/guidelines.xhtml

2008 Malawi census data. Each sick child was linked to the geographic centroid of the associated household's EA using EA codes provided by the Malawi National Statistical Office. SPA data include exact location of each facility. There was limited empirical evidence from Malawi regarding where children were taken to seek treatment for their illness. In this study, rather than assessing bypassing, we focused on whether having a high quality facility nearby promotes household utilization of sick child health services. We matched each sick child and his/her household to the single nearest facility, based on travel distance by road to the EA centroid of the sick child's household. To calculate the geographic distance, we executed four steps: first identified the closet 8 facilities providing sick child healthcare by using the geocoordinates for facilities (exact location) and household clusters (centroid); second, the road distance was calculated between cluster centroid and each facility based on the Google Maps API, including linear distance to nearest road where coordinates are not directly on a road (road type is not incorporated in this calculation); third, we replaced road distance with linear distance if road distance can not be calculated (e.g., if there is no road on an island) or if road distance is less than linear distance; lastly, we identified the facility with minimum distance to the cluster. Steps 1, 3, and 4 were executed in Stata, and Step 2 was run in Python 3.6.1. Based on prior studies suggesting that household distance to nearest health facility in Malawi is rarely greater than 50 km (34-36), we excluded children whose nearest facility was over 50 km away.

Descriptive analyses of facility quality for sick child healthcare were first performed. We used logistic regression analyses of utilization of sick child healthcare on the quality index and then adjusted for the covariates of interest. We used clustered standard errors to account for the non-independence of observations within EAs. To understand which element of the overall

For peer review only - http://bmjopen.bmj.com/site/about/guidelines.xhtml

quality served best as a predictor for household utilization of sick child healthcare, we separated overall facility quality into structural and process quality.

We further conducted a series of sensitivity analyses to understand the robustness of our results. To understand whether the best performing facility may be more influential than the nearest facility, as long as it's still relatively accessible, we matched the household with the best performing facility within the buffer zone of a 5-km radius (direct distance from the household's EA centroid). Additionally, to test the sensitivity of catchment area definitions, we continued using the best performing facility to match with the households, but modified the original 5-km buffer zones to 10-km and 20-km direct distance radius from the household's EA centroid.

Statistical analyses were run in Stata (version 14.1), mapping was done using QGIS Version 2.18 (Free Software Foundation, Massachusetts), and geographic distances (e.g., road traveling distances) were calculated based on Google Maps using Python 3.6.1.

RESULTS

Participants

In the 2013 SPA, 977 of a total of 1,066 surveyed health facilities (response rate: 92%) completed the assessment. Among these 977 facilities, 920 facilities (94%) offered sick child health services. Among these 920 facilities, 746 facilities had observations of sick child healthcare; process quality indicators were imputed for the 174 facilities that offer sick child services but did not have any observations of care. Completeness of each indicator is shown in Appendix Table 1. There was no missingness for infrastructure, but minimal for equipment, essential supplies and medications, substantial for supervision (up to 14%), and moderate for process quality (up to 19%).

For peer review only - http://bmjopen.bmj.com/site/about/guidelines.xhtml

The 2013 MICS dataset included 18,981 children under five years of age with completed caretaker interviews (response rate: 98%). Among these responding households, 52% of children (n=9,811) were reported by their caretakers as having symptoms of diarrhea (n=4,419, 45%), ARI (n=1,438, 15%), or fever (n=7,118, 73%). Among the 9,811 children who had illnesses, 6,679 children (68%) sought care at facilities (hospitals, health centers/clinics, or health posts/dispensaries).

In our main matching strategy, 110 of the 9,811 sick children were dropped due to the EAs for which locations are not available. Then, each of the 9701 sick children was matched to the child's single nearest facility of the 920 health facilities providing sick child care based on road traveling distance to the EA centroid of the sick child's household. In this step, 352 health facilities were dropped. Therefore, our main matching strategy yielded an analytical sample of 568 health facilities providing sick child healthcare services and 9,701 children who were reported by their caregivers as having illness of diarrhea, fever, or ARI. A total of 8,363 (86%) sick child consultations were actually observed in these selected facilities.

Descriptive Data

Table 1 detailed characteristics of health facilities in our main analytic sample. Among the health facilities that were included in the main analytic sample, health centers (57%) and clinics (26%) were the most common. The bulk of health facilities had clinical officers as the highest level of provider present (79%). The number of facilities located in rural areas was about four-fold those in urban settings, while about half were managed by government authority. Figure 1 shows the geographic distribution of all SPA health facilities and the ones included in

For peer review only - http://bmjopen.bmj.com/site/about/guidelines.xhtml

our analytic sample, as well as the population density in the enumeration areas. Most facilities were located in the densely populated areas of Malawi.

Figure 1 to be inserted here.

For peer review only

For peer review only - http://bmjopen.bmj.com/site/about/guidelines.xhtml

321 **Table 1. Facility characteristics and quality performance on sick child healthcare services**
322 **in the analytic sample (Main Model: n=568)** £

	**All facilities (568)**	
	n or mean	% or SD
Rural§	445	78.4
Urban	123	21.6
Public¶	300	53.0
Private	268	47.0
Facility type		
Hospital	75	13.2
Health center	323	56.9
Clinic	145	25.5
Health post	3	0.5
Other (dispensaries)	22	3.9
Highest clinician on site		
Medical doctor	60	10.6
Registered nurse	8	1.4
Enrolled nurse	38	6.7
Assistant medical officer	9	1.6
Clinical officer	448	78.9
Other health professional	5	0.9
Overall Quality Performance (mean, SD)		
Structural quality	0.62	0.14
Process quality	0.33	0.14
Overall quality	0.48	0.10

For peer review only

For peer review only - http://bmjopen.bmj.com/site/about/guidelines.xhtml

£ In the main model (n-568), the analysis was restricted to facilities offering sick child healthcare that matched (within 50km) to a household sampled in the MICS with a recently ill child.

.

§ Facility is in rural area.

¶ Facility is managed by government authority.

Figures 2 and 3 detail the facility performance on a structural and process quality index, respectively. The average structural quality score for health facilities in Malawi providing sick child curative care services was 0.62 (SD: 0.14, range: 0.20- 0.97) and the average process quality score was 0.33 (SD: 0.14, range: 0.04- 0.78). The average overall quality score was 0.48 (SD: 0.10, range: 0.19- 0.90). Facilities were commonly equipped with basic infrastructure, such as client waiting rooms and general facility cleanliness, while they still lacked light sources, electricity, toilet, ambulance, and computer or internet access. Health facilities achieved high level performance on essential supplies and medication readiness, while about forty percent (40%) had sick child health service room infection control supplies. Within the staffing and management domain, nearly all facilities reported employing some form of supervision, while a modest number performed well in routine quality assurance or else received inadequate staff training on IMCI child health services. Reporting client opinions was extremely rare. With the exception of high percentages in process quality indicators achieved for fever, coughing, and temperature examination, all other indicators performed relatively modestly. The poorest indicator was providing counseling and examination for danger signs, edema, maternal-infant transmission of HIV, and ear pain.

Figure 2 to be inserted here.

Figure 3 to be inserted here.

Table 2 provides characteristics of sick children in the analytic sample, of whom, 6,679 children (69%) sought care at facilities during their illness, while 3,022 (31%) did not seek care. The average age of sick children was 29 months (SD: 16 months), with 16% younger than one

For peer review only - http://bmjopen.bmj.com/site/about/guidelines.xhtml

year old. Half (50%) of the sick children were girls. The bulk of sick children had a perceived symptom of fever (64%), with ARI the least common (15%). The average road traveling distance to the nearest facility was about 5.8 km (SD: 4.7 km; median: 4.7 km).

For peer review only

For peer review only - http://bmjopen.bmj.com/site/about/guidelines.xhtml

353 **Table 2. Characteristics of sick child in the analytic sample (Main model: n=12,258)**

	Total sick children (n=9,701)		Sought for care at facilities during illness (n= 6,679)	No care-seeking during illness (n= 3,022)
**Variable**	**n**	**%[§]**	**n(%[¶])**	**n(%[¶])**
**Child age (in months)**				
**≤ 2 months**	162	1.7	88(54.3%)	74(45.7%)
**2 months- 1 year**	1,537	15.8	1052(68.4%)	485(31.6%)
**1 year - 5 years**	8,002	82.5	5539(69.2%)	2463(30.8%)
**Child sex**				
**Male**	4,870	50.2	3420(70.2%)	1450(29.8%)
**Female**	4,831	49.8	3259(67.5%)	1572(32.5%)
**Child sickness type**				
**Diarrhea**	2,097	21.6	1284(61.2%)	813(38.8%)
**Fever**	6,185	63.8	4352(70.4%)	1833(29.6%)
**ARI**	1,419	14.6	1043(73.5%)	376(26.5%)
**Mother's education**				

For peer review only - http://bmjopen.bmj.com/site/about/guidelines.xhtml

**None**	1,287	13.2	817(63.5%)	470(36.5%)
**Primary**	7,016	72.3	4828(68.8%)	2188(31.2%)
**Secondary or above**	1,392	14.3	1031(74.1%)	361(25.9%)
**Household wealth quintile**				
**Q1 (poorest)**	2,380	24.5	1605(67.4%)	775(32.6%)
**Q2**	2,227	23	1515(68.0%)	712(32.0%)
**Q3**	2,140	22.1	1472(68.8%)	668(31.2%)
**Q4**	1,703	17.6	1205(70.8%)	498(29.2%)
**Q5 (richest)**	1,251	13	882(70.5%)	369(29.5%)
**Road Traveling Distance (km) to nearest facility mean (SD, median)**	5.77(4.7, 4.8)		5.66(4.7, 4.7)	6.00(4.7, 4.9)

354 § Column percentages.
355 ¶ Row percentages.
356 £ n=9,701, number of children who were perceived as sick in last two weeks by their caretakers that plausibly matched with their households' single nearest sick child health service facility in SPA. In the main model (n-568), the analysis was restricted to facilities offering sick child healthcare that matched (within 50km) to a household sampled in the MICS with a recently ill child.

For peer review only - http://bmjopen.bmj.com/site/about/guidelines.xhtml

359 **Main findings**

360 Table 3 show results of the multivariable logistic regression models of the association between quality of care and sick child healthcare service utilization in Malawi health facilities. Model 1 and Model 4 represent the base and fully-specified models, respectively. Models 2 and 3 represent models adjusting for individual factors. Model 5 represents the fully specified model with the quality index separated into structural inputs and process quality.

365 The overall quality index of structural and process quality was a significant predicator of utilizing formal health facilities for sick child healthcare services in Malawi. In the fully specified model (Model 4), the odds of utilizing formal health facilities increases by 66% (AOR: 1.66, 95% CI: 1.04, 2.63) for the overall quality change from an absolute minimum of 0 to maximum of 1 (perfect quality). The magnitude of the effect increases slightly when adjusting for the control variables, and remains significant in all models (Model 1-4). When separating the overall quality index into structural inputs and the process quality in the full specified model (Model 5), structural quality was a significant predictor (AOR: 1.33, 95% CI: 0.95, 1.87) while process quality was positively but not significantly associated with utilization (AOR: 1.25, 95% CI: 0.91, 1.72). The overall quality remained as a significant predicator for utilizing health facilities for sick child healthcare in the three sensitivity analyses (SA) shown in Appendix Table 2. These SA findings supported the association between facility quality and sick child healthcare utilization even when using a large catchment area.

378 A child's type of illness and mother's education were found to be significant individual-level predictors of sick child healthcare utilization. For children's illness type, in the fully specified model (Model 4) the results suggest higher odds of utilizing health facilities for children with a reported fever (AOR: 1.17, 95% CI: 1.03, 1.34) and symptoms of ARI

For peer review only - http://bmjopen.bmj.com/site/about/guidelines.xhtml

(AOR:1.78, 95% CI: 1.53, 2.06), compared to those who were perceived to have diarrhea. This indicates that the severity of childhood illness is associated with motivating caregivers to utilize health facilities. For mother's education, the results suggest a gradient with increasing odds of utilizing health facilities as the level of the mother's education increases. Compared to mothers who did not have primary education, mothers who had primary education and those had secondary or higher education had 30% (AOR: 1.30, 95% CI: 1.13, 1.49) and 65% (AOR:1.65, 95% CI: 1.38, 1.97) higher odds of utilizing health facilities, respectively, after controlling for other factors.

For peer review only

For peer review only - http://bmjopen.bmj.com/site/about/guidelines.xhtml

Table 3. Regression results for the association between sick child healthcare utilization and the overall quality (structural and process quality) of health service facility in Malawi. £

**Main Models**	(1)		(2)		(3)		(4)		(5)	
VARIABLES	Odds ratio (P-value)	95% CI¶	Adjusted odds ratio (P-value)	95% CI	Adjusted odds ratio (P-value)	95% CI	Adjusted odds ratio (P-value)	95% CI	Adjusted odds ratio (P-value)	95% CI
Overall quality	1.53(0.08)	(0.96, 2.43)	1.61 (0.05)	(1.01, 2.56)	1.67 (0.03)	(1.05, 2.65)	1.66(0.03)	(1.04, 2.63)	-	
Structural quality			-		-		-		1.33(0.10)	(0.95, 1.87)
Process quality			-		-		-		1.25(0.17)	(0.91, 1.72)
Child age										
(Ref: ≤ 2 months)										
2 months- 1 year			0.99(0.90)	(0.88, 1.12)	1.00 (1.00)	(0.89, 1.13)	1.00(1.00)	(0.89, 1.13)	1.00(1.00)	(0.89, 1.13)
1 year- 5 years			1.98(0.00)	(1.45, 2.72)	1.99 (0.00)	(1.45, 2.73)	1.99(0.00)	(1.45, 2.73)	1.99(0.00)	(1.46, 2.73)
Child sex										
(Ref: Male)										
Female			0.88 (0.00)	(0.80, 0.95)	0.88 (0.00)	(0.80, 0.96)	0.88(0.00)	(0.80, 0.96)	0.88(0.00)	(0.80, 0.96)
Child sickness type										
(Ref: diarrhea)										
Fever			1.17 (0.02)	(1.02, 1.33)	1.17 (0.02)	(1.03, 1.34)	1.17(0.02)	(1.03, 1.34)	1.17(0.02)	(1.03, 1.34)

For peer review only

For peer review only - http://bmjopen.bmj.com/site/about/guidelines.xhtml

ARI			1.77 (0.00)	(1.53, 2.06)	1.78 (0.00)	(1.54, 2.06)	1.78(0.00)	(1.53, 2.06)	1.79(0.00)	(1.53, 2.06)
Mother education (Ref: No education)										
Primary					1.29 (0.00)	(1.12, 1.49)	1.29(0.00)	(1.13, 1.49)	1.30(0.00)	(1.13, 1.49)
Secondary or Higher					1.65 (0.00)	(1.38, 1.97)	1.65(0.00)	(1.38, 1.97)	1.65(0.00)	(1.38, 1.97)
Household Wealth Quintile (Ref: Q1 poorest)										
Q2					0.91 (0.24)	(0.07)	0.92(0.38)	(0.77, 1.10)	0.92(0.38)	(0.77, 1.10)
Q3					0.96 (0.60)	(0.08)	0.98(0.82)	(0.82, 1.17)	0.98(0.82)	(0.82, 1.17)
Q4					0.99 (0.87)	(0.08)	1.01(0.92)	(0.84, 1.21)	1.01(0.91)	(0.84, 1.21)
Q5					1.00 (0.96)	(0.08)	1.02(0.84)	(0.85, 1.22)	1.01(0.83)	(0.85, 1.22)
Household Rural Residence							0.95(0.57)	(0.80, 1.13)	0.95(0.56)	(0.80, 1.13)
Observations	9,701		9,701		9,695		9,695		9,695	

*** p<.01, ** p<.05, * p<.1

£ In the main model (n-568), the analysis was restricted to facilities offering sick child healthcare that matched (within 50km) to a household sampled in the MICS with a recently ill child.

For peer review only - http://bmjopen.bmj.com/site/about/guidelines.xhtml

395 ¶ 95% CI: 95% Confidence Intervals

For peer review only

For peer review only - http://bmjopen.bmj.com/site/about/guidelines.xhtml

DISCUSSION

In this paper, we sought to determine the influence of facility-based quality of healthcare on care-seeking behaviors of Malawian parents faced with childhood illness. We found that facility quality is an important predictor of parental decision-making regarding care-seeking for illnesses such as diarrhea, fever, and ARI among children under five years, using national data from Malawi. Our sensitivity analyses varying the catchment areas consistently supported this association.

We found that about 69% of surveyed caregivers for sick children in our analytic sample utilized facility-based healthcare services in Malawi, reflecting a similar prevalence of care-seeking reported by the most recent two Malawi DHS surveys (7, 37). Although Malawi has been one of the top three countries with highest prevalence of care-seeking for children with reported fever, diarrhea, or symptoms of ARI in African maternal and child health (MCH) priority countries, care-seeking for sick under-five children was still not optimal given the target of ending preventable deaths of under five children by 2030 in Malawi Health Sector Strategic Plan (HSSP).

Our findings confirmed past research showing major gaps in service readiness and provider competence in sick child healthcare. Like this work, others have noted poor provider performance, with fewer than half of clinical actions completed (e.g., taking patient history, examination, and counseling) (18, 20). Moreover, guideline adherence was low, with a high rate of missed diagnosis among children (four out of every five children) with pneumonia (38).

Our work advances on prior research in several ways. Past studies have largely focused on other factors in determining a caregiver's appropriate care-seeking for childhood illness, such as socio-demographic and household characteristics, social cultural factors, geographic access,

For peer review only - http://bmjopen.bmj.com/site/about/guidelines.xhtml

travel time, health facility fees, insurance, health worker densities, etc (39-43). Most prior studies on quality care for sick child healthcare services were specific evaluations of quality improvement interventions, such as IMCI community health worker programs (44-46). Few studies have examined the relationship of quality and healthcare utilization on a national scale with regard to childhood illness in LMICs, with only one publication from Kenya attempting to construct the relationship between patient-perceived quality and the low attendance at the maternal and child health services using local district data.

When disaggregating our quality measure into structural and process components, we found that structural readiness (facility infrastructure, equipment, and staff) was a significant predictor of utilization, while process quality (adherence to medical guidelines) was positively but not significantly predictive of utilization. This finding suggests that individuals (caregivers) may weigh visible health facility characteristics (such as presence of client waiting room, general facility cleanness, equipment, drugs, etc.) more than they weigh the actual clinical care service experience. Because of the asymmetry of information between providers and patients in the healthcare market, patients do not have full knowledge about what constitutes good quality; in this case, they may not be fully aware of the recommended components of clinical assessment for their child (47).

This study had several strengths. First, the availability of exact spatial location data of the SPA health facilities and all EAs for the MICS from the 2008 Malawi census data provided a unique opportunity to examine health system and population data together in concert to answer a policy relevant question. By contrast, DHS household locations are displaced up to 5km in rural areas, which precludes accurate matching to nearest facility. Second, we relied on WHO SARA

For peer review only - http://bmjopen.bmj.com/site/about/guidelines.xhtml

and IMCI guidelines to define quality measures. Furthermore, multiple sensitivity analyses confirmed that the findings were consistent across different facility matching specifications.

However, this study has several limitations. First, this study is based on cross-sectional data and is therefore unable to address causal claims. Second, given the available Malawi healthcare utilization data (2013 MICS), we chose the most clinically relevant questions (variables) to indicate the demand by under-five children with sickness of diarrhea, malaria, or pneumonia. Our study focused on understanding the influence of quality on caregivers' decisions to utilize health facilities for their sick child. However, no patient outcome data were available, which prevented investigation of the linkage between facility quality and patient outcomes. Third, due to the data limitations, we extracted the quality index items available from the Malawi SPA dataset with reliance on the WHO SARA and IMCI, and therefore this analysis focused on specific health facility characteristics and interviewer-observed clinical quality service, which however do not assess individual perceptions of healthcare quality, nor address the patient-provider interpersonal quality of care. Additionally, we acknowledged the common challenge of classifying childhood illness based on survey data in LMICs, (48, 49) thereby our research was unable to capture the sufficient specificity on illness severity and identify all children with these symptoms who actually require formal healthcare at health facilities. Furthermore, in our multiple imputation strategy, we assumed that missingness of the health facility which provided sick child healthcare but had 0 observations within a stratum like rural dispensaries is random. Our assumption was not that the missingness of these observations is randomly distributed across hospitals and dispensaries. Therefore, we included four covariates (facility type, facility managing authority, districts, and rural/urban) in the multiple imputation. Moreover, in the real world, caretaker decisions in seeking care for a sick child, as well as where to seek healthcare,

For peer review only - http://bmjopen.bmj.com/site/about/guidelines.xhtml

can be influenced by their perception of the quality of child health services at facilities recently visited. Our study used the facility-based patient data that captures the nearest facility to the patient's household and we were not able to identify the previous facility which the patient visited. Given literature from other nations with comparable settings, in their first action, caregivers would seek healthcare at the nearest facility upon recognizing the child's symptom; however, they would choose to bypass their nearest facility when it lacked diagnostic equipment, drugs, and skilled health workers, or had poor services. (22, 50-52) . In this study, we were not able to examine the influence of quality of the previous facility in relation to the caregiver's next health services facility choice, although these types of associations have been beneficial in prior studies in developed countries (53). Thus, the matching strategy of linking a sick child to a health facility may not completely reflect actual behavior.

Policy Implications

Our findings have several implications for policy and future research. Our study provides strong and direct empirical evidence that better quality health facilities are associated with increased healthcare utilization for childhood illness, which, if care is sufficiently competent, can improve odds of survival from treatable conditions. As our findings suggested, the government of Malawi may consider the improvement of the health facility structure, including equipment, essential supplies, drug storage and availability, room cleanness and infection control. Visible improvements are most likely to attract caregivers in utilization of the health facilities when they perceive childhood illness. However, beyond driving utilization, provider clinical competence needs to improve as well if visits are to be converted into better health. Given the low level of provider performance we and others have documented, this will require structure system reforms,

For peer review only - http://bmjopen.bmj.com/site/about/guidelines.xhtml

potentially including updating pre-service education and re-organizing where healthcare is delivered for maximum gains (6).

Future research is needed to validate and extend these findings in other country settings. Malawi has been a leader in sub-Saharan Africa, demonstrating strong political will to implement evidence-based interventions that can improve maternal and child health. The availability of the geocoded health facilities and population data enabled us to match the health system facility survey data and the population data in. Other countries could take similar measures to permit matching of health system and household data to obtain better insights in how health systems influence health and care seeking. These measures would aid in policy determinations, to evaluate whether services supplied by the health system can actually meet with the demands of the population, and quality of care can satisfy the population's needs and promote patient care-seeking behaviors that reduce preventable deaths. In addition, the facility quality index needs to be validated in different country settings, encompassing higher mortality burdens and different health system capacities, to strengthen the generalizability of the results.

As an important component of human capital, health can contribute positively to a nation's economic development (54). At the same time, achieving an excellent state of health is an intrinsic part of the goals of social development, as well as an essential factor in an individual's well-being. Good quality healthcare is thus both an intrinsic good that can promote health outcomes as well as a driver of utilization; to achieve these ends both structures and processes of care need to improve.

For peer review only - http://bmjopen.bmj.com/site/about/guidelines.xhtml

Acknowledgements

Earlier versions of this paper were presented during several seminars at the Harvard Global Health Institute, the Harvard T.H. Chan School of Public Health, and the Fifth Global Symposium on Health Systems Research, where valuable comments were received from seminar participants, including Stéphane Verguet, Michael Reich and Sara Singer. The authors thank Dennis Lee and Fei Carnes for assistance with geographic linking. The authors also acknowledge Humphreys Nsona for his input on the initial study idea and assistance with the geospatial data.

Contributors: MEK conceptualized the study. HHL and LL curated data. LL conducted the formal analysis. HHL contributed to study design. LL visualized the data results and wrote the original draft. LL, HHL, MJ, and MEK reviewed and contributed to the editing of the manuscript. All authors approved the final manuscript submitted.

Funding: This research received no specific grant from any funding agency in the public, commercial or not-for-profit sectors.

Competing Interests Statement: None declared.

Patient Consent: Patients and the public were not involved in the research design or planning of the study therefore the patient consent was not required.

Ethical approval: The Harvard University Human Research Protection Program deemed this analysis of secondary data exempt from human subject review.

Data Sharing Statement: SPA data files are available from the DHS Program (http://dhsprogram.com/whatwedo/survey/survey-display-424.cfm). MICS survey are available from UNICEF (http://mics.unicef.org/surveys); cluster sampling details were provided on request from the Malawi National Statistical Office.

For peer review only

For peer review only - http://bmjopen.bmj.com/site/about/guidelines.xhtml

References

1. The United Nations Millennium Development Goals. [cited 2017 September 20]. Available from: http://www.un.org/millenniumgoals/.
2. The United Nations Sustainable Development Goals. Available from: http://www.un.org/sustainabledevelopment/sustainable-development-goals/.
3. World Health Organization. World health report 2013: Research for universal health coverage. 2013.
4. United Nations General Assembly. Ga/11326. [cited 2017 October 20]. Available from: http://www.un.org/en/ga/67/resolutions.shtml.
5. Berwick DM, Kelley E, Kruk ME, et al. Three global health-care quality reports in 2018. The Lancet. 2018;392(10143):194-5.
6. Kruk ME, Gage AD, Arsenault C, et al. High-quality health systems in the sustainable development goals era: Time for a revolution. The Lancet Global Health. 2018;6(11):e1196-e252.
7. National Statistical Office (NSO) [Malawi] and ICF. Malawi demographic and health survey 2015-16. Zomba, Malawi, and Rockville, Maryland, USA. NSO and ICF: 2017.
8. Central Statistical Agency (CSA) [Ethiopia] and ICF. Ethiopia demographic and health survey 2016. Addis Ababa, Ethiopia, and Rockville, Maryland, USA: CSA and ICF: 2016.
9. Ministry of Health CD, Gender, Elderly and Children (MoHCDGEC) [Tanzania Mainland], Ministry of Health (MoH) [Zanzibar], National Bureau of Statistics (NBS), Office of the Chief Government Statistician (OCGS), and ICF. Tanzania demographic and health survey and malaria indicator survey (tdhs-mis) 2015-16. Dar es Salaam, Tanzania, and Rockville, Maryland, USA: MoHCDGEC, MoH, NBS, OCGS, and ICF: 2016.
10. Kenya National Bureau of Statistics MoHK, National AIDS Control Council/Kenya, Kenya Medical Research Institute, National Council for Population and Development/Kenya, and ICF International. Kenya demographic and health survey 2014. Rockville, MD, USA: Kenya National Bureau of Statistics, Ministry of Health/Kenya, National AIDS Control Council/Kenya, Kenya Medical Research Institute, National Council for Population and Development/Kenya, and ICF International: 2015.
11. United Nations Development Porgramme (UNDP). 2014 millennium development goal report for malawi. United Nations Development Programme, New York: 2014.
12. The Demographic and Health Surveys. Malawi service provision assessment (spa) 2013-14.
13. National Statistical Office/Malawi and Macro International. Malawi demographic and health survey 1992. Zomba, Malawi: National Statistical Office/Malawi and Macro International: 1994.
14. Sobel HL, Huntington D, Temmerman M. Quality at the centre of universal health coverage. Health Policy and Planning. 2015;31(4):547-9.
15. Hanefeld J, Powell-Jackson T, Balabanova D. Understanding and measuring quality of care: Dealing with complexity. Bulletin of the World Health Organization. 2017;95(5):368.
16. Kruk ME, Larson E, Twum-Danso NA. Time for a quality revolution in global health. The Lancet Global Health. 2016;4(9):e594-e6.
17. Abera Abaerei A, Ncayiyana J, Levin J. Health-care utilization and associated factors in gauteng province, south africa. Global health action. 2017;10(1):1305765.
18. Leslie HH, Ndiaye Y, Kruk ME. Effective coverage of primary care services in eight high-mortality countries. BMJ Global Health. 2017;2(3):e000424.

For peer review only - http://bmjopen.bmj.com/site/about/guidelines.xhtml

19. Gera T, Shah D, Garner P, et al. Integrated management of childhood illness (imci) strategy for children under five: Effects on death, service utilisation and illness. Cochrane Database of Systematic Reviews. 2012(9).
20. Kruk ME, Gage AD, Mbaruku GM, et al. Content of care in 15,000 sick child consultations in nine lower-income countries. Health services research. 2018.
21. Uwemedimo OT, Lewis TP, Essien EA, et al. Distribution and determinants of pneumonia diagnosis using integrated management of childhood illness guidelines: A nationally representative study in malawi. BMJ global health. 2018;3(2):e000506.
22. Audo M, Ferguson A, Njoroge P. Quality of health care and its effects in the utilisation of maternal and child health services in kenya. East African Medical Journal. 2005;82(11).
23. Creanga AA, Gullo S, Kuhlmann AKS, et al. Is quality of care a key predictor of perinatal health care utilization and patient satisfaction in malawi? BMC pregnancy and childbirth. 2017;17(1):150.
24. Kruk ME, Mbaruku G, McCord CW, et al. Bypassing primary care facilities for childbirth: A population-based study in rural tanzania. Health Policy and Planning. 2009;24(4):279-88.
25. Gage AD, Leslie HH, Bitton A, et al. Assessing the quality of primary care in haiti. Bulletin of the World Health Organization. 2017;95(3):182.
26. World bank data indicators [Internet]. [cited March 03, 2017]. Available from: http://data.worldbank.org/.
27. National Statistical Office. Malawi mdg endline survey 2014. Zomba, Malawi: National Statistical Office; 2015
28. UNICEF, WHO, World Bank, UN-DESA Population Division. Levels and trends in child mortality report 2017. New York: United Nations Children's Fund, 2017.
29. Kruk ME, Pate M, Mullan Z. Introducing the lancet global health commission on high-quality health systems in the sdg era. The Lancet Global Health. 2017;5(5):e480-e1.
30. The Institute of Medicine. Crossing the quality chasm: A new health system for the 21st century. 2001
31. Donabedian A. Evaluating the quality of medical care. Milbank Quarterly. 2005;83(4):691-729.
32. Campbell SM, Roland MO, Buetow SA. Defining quality of care. Social science & medicine. 2000;51(11):1611-25.
33. Honaker J, King G, Blackwell M. Amelia ii: A program for missing data. Journal of statistical software. 2011;45(7):1-47.
34. Ewing VL, Lalloo DG, Phiri KS, et al. Seasonal and geographic differences in treatment-seeking and household cost of febrile illness among children in malawi. Malaria Journal. 2011;10(1):32.
35. Karra M, Fink G, Canning D. Facility distance and child mortality: A multi-country study of health facility access, service utilization, and child health outcomes. International Journal of Epidemiology. 2016;46(3):817-26.
36. Malawi National Statistical Office. Malawi demographic and health survey 1992 National Statistical Office, Zomba, Malawi, 1992.
37. National Statistical Office (NSO) and ICF Macro. Malawi demographic and health survey 2010. Zomba, Malawi, and Calverton, Maryland, USA: NSO and ICF Macro: 2011.

For peer review only - http://bmjopen.bmj.com/site/about/guidelines.xhtml

38. Omolara T. Uwemedimo, Todd P. Lewis, Elsie A. Essien, et al. Distribution and determinants of pneumonia diagnosis using integrated management of childhood illness guidelines: A nationally representative study in malawi. BMJ Global Health. Forthcoming.
39. Abdulkadir MB, Abdulkadir ZA. A cross-sectional survey of parental care-seeking behavior for febrile illness among under-five children in nigeria. Alexandria Journal of Medicine. 2017;53(1):85-91.
40. Noordam AC, Carvajal-Velez L, Sharkey AB, et al. Correction: Care seeking behaviour for children with suspected pneumonia in countries in sub-saharan africa with high pneumonia mortality. PLoS One. 2015;10(4):e0126997.
41. Shaw B, Amouzou A, Miller NP, et al. A qualitative exploration of care-seeking pathways for sick children in the rural oromia region of ethiopia. BMC health services research. 2017;17(1):184.
42. Chibwana AI, Mathanga DP, Chinkhumba J, et al. Socio-cultural predictors of health-seeking behaviour for febrile under-five children in mwanza-neno district, malawi. Malaria journal. 2009;8(1):219.
43. Hjortsberg C. Why do the sick not utilise health care? The case of zambia. Health economics. 2003;12(9):755-70.
44. Cardemil CV, Gilroy KE, Callaghan-Koru JA, et al. Comparison of methods for assessing quality of care for community case management of sick children: An application with community health workers in malawi. The American journal of tropical medicine and hygiene. 2012;87(5_Suppl):127-36.
45. Miller NP, Amouzou A, Tafesse M, et al. Integrated community case management of childhood illness in ethiopia: Implementation strength and quality of care. The American journal of tropical medicine and hygiene. 2014;91(2):424-34.
46. Nsona H, Mtimuni A, Daelmans B, et al. Scaling up integrated community case management of childhood illness: Update from malawi. The American journal of tropical medicine and hygiene. 2012;87(5_Suppl):54-60.
47. Arneill AB, Devlin AS. Perceived quality of care: The influence of the waiting room environment. Journal of Environmental Psychology. 2002;22(4):345-60.
48. Munos MK, Stanton CK, Bryce J. Improving coverage measurement for reproductive, maternal, neonatal and child health: Gaps and opportunities. Journal of global health. 2017;7(1).
49. Arnold F, Khan SM. Perspectives and implications of the improving coverage measurement core group's validation studies for household surveys. Journal of global health. 2018;8(1).
50. Kruk ME, Hermosilla S, Larson E, et al. Bypassing primary care clinics for childbirth: A cross-sectional study in the pwani region, united republic of tanzania. Bulletin of the World Health Organization. 2014;92(4):246-53.
51. Kahabuka C, Kvåle G, Moland KM, et al. Why caretakers bypass primary health care facilities for child care-a case from rural tanzania. BMC health services research. 2011;11(1):315.
52. Lohela TJ, Campbell OM, Gabrysch S. Distance to care, facility delivery and early neonatal mortality in malawi and zambia. PLoS One. 2012;7(12):e52110.
53. Jung K, Feldman R, Scanlon D. Where would you go for your next hospitalization? Journal of Health Economics. 2011;30(4):832-41.
54. Bloom DE, Canning D, Sevilla J. The effect of health on economic growth: A production function approach. World development. 2004;32(1):1-13.

For peer review only - http://bmjopen.bmj.com/site/about/guidelines.xhtml

Figure 1. Distribution of health facilities in Malawi relative to MICS enumeration areas (EAs).

Figure 2. Performance on facility structural quality index: percentage of facilities with key resources and services (n=568).

Figure 3. Performance on facility process quality index: percentage of facilities with key resources and services (n=568).

For peer review only

For peer review only - http://bmjopen.bmj.com/site/about/guidelines.xhtml

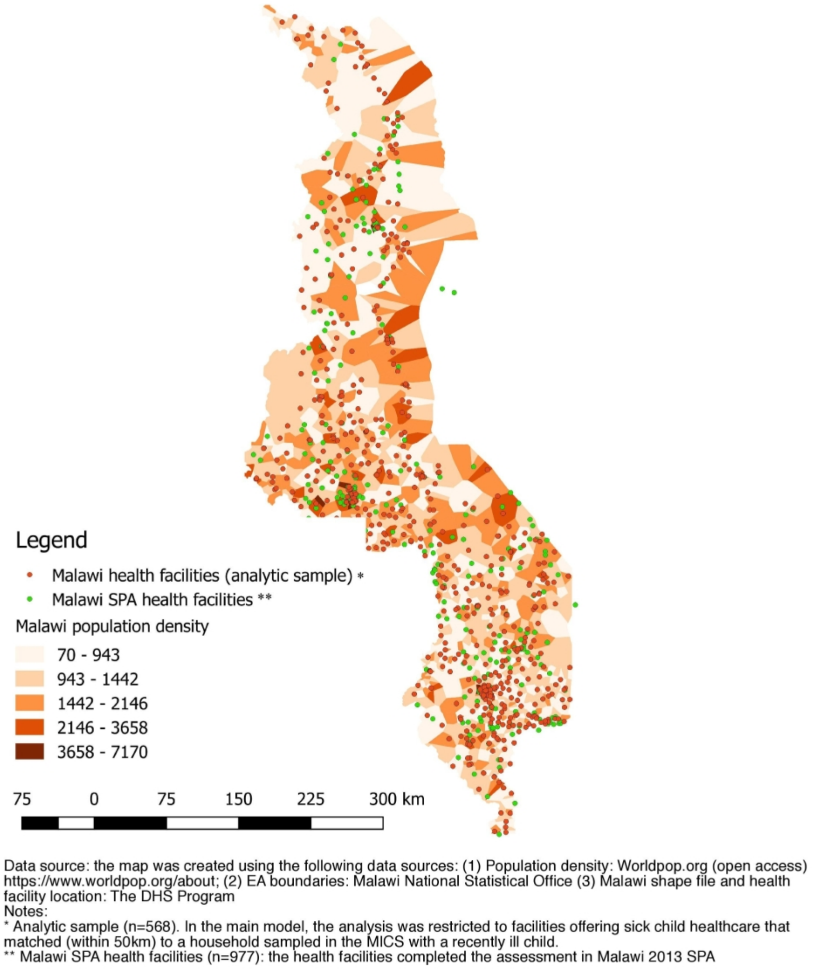

Figure 1. Distribution of health facilities in Malawi relative to MICS enumeration areas (EAs).

215x279mm (300 x 300 DPI)

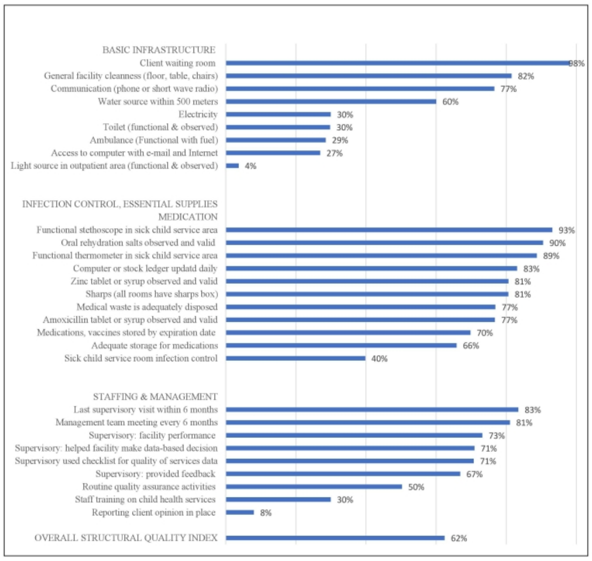

Figure 2. Performance on facility structural quality index: percentage of facilities with key resources and services (n=568).

338x190mm (300 x 300 DPI)

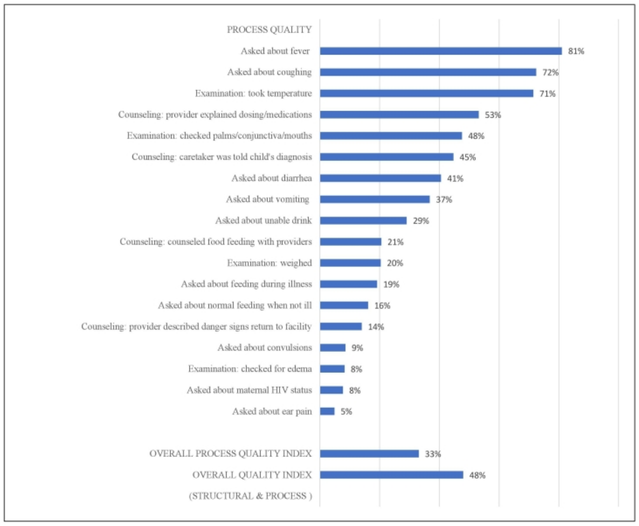

Figure 3. Performance on facility process quality index: percentage of facilities with key resources and services (n=568).

338x190mm (300 x 300 DPI)

Appendix Table 1: Completeness of indicators for facilities† (n=920) and observations

Variable	% Missing	% Completed
Water (improved water source round within 500 meters)	0	100
Ambulance (Functional with fuel)	0	100
Electricity	0	100
Communication (phone or short wave radio)	0	100
Toilet (functional & observed)	0	100
General facility cleanness (floor, table, chairs)	0	100
Client waiting room	0	100
Access to computer with e-mail and Internet	0	100
Light source (functional & observed)	0	100
Sick child health service room infection control	0	100
Sharps (all rooms have sharps box)	0.33	99.67
Medical waste is adequately disposed	0.43	99.57
Functional thermometer in sick child health service area	0	100
Functional stethoscope in sick child health service area	0	100
Oral rehydration salts in pharmacy or sick child health service area	0	100
Amoxicillin tablet or syrup observed and valid	0.65	99.35
Zinc tablet or syrup observed and valid	0.65	99.35
Medications, vaccines stored according to expiration date	11.96	88.04
Adequate storage for medications	0	100
Computer or stock ledger updated daily	0	100
Staff training on child health services	0	100
Last supervisory visit within 6 months	0	100
Management team meeting every 6 months	0.65	99.35
Supervisory used checklist for quality of health services data	13.70	86.30

For peer review only - http://bmjopen.bmj.com/site/about/guidelines.xhtml

Supervisory: facility performance	13.70	86.30
Supervisory: helped facility make data-based decision	13.70	86.30
Supervisory: provided feedback	13.70	86.30
Reporting client opinion in place	1.20	98.8
Routine quality assurance activities	0.87	99.13
Asked about vomiting	18.91	81.09
Asked about unable to drink	18.91	81.09
Asked about convulsions	18.91	81.09
Asked about normal feeding when not ill	18.91	81.09
Asked about maternal HIV status	18.91	81.09
Asked about feeding during illness	18.91	81.09
Asked about fever	18.91	81.09
Asked about ear pain	18.91	81.09
Asked about diarrhea	18.91	81.09
Asked about coughing	18.91	81.09
Examination: weighed	18.91	81.09
Examination: took temperature	18.91	81.09
Examination: checked palms/conjunctiva/mouths	18.91	81.09
Examination: checked for edema	18.91	81.09
Counseling: provider explained dosing/medications	19.24	80.76
Counseling: counseled food feeding with providers	18.91	81.09
Counseling: caretaker was told child's diagnosis	18.91	81.09
Counseling: provider described danger signs return to facility	18.91	81.09

[†] A total of 920 health facilities from 2013 Malawi SPA that provided child curative care was included in this study.

For peer review only - http://bmjopen.bmj.com/site/about/guidelines.xhtml

Appendix Table 2. Sensitivity analyses model results for the association between sick child healthcare utilization and the overall quality (structural and process quality) of health service facility in Malawi.

**Model**	**Adjusted results** †		**N**
	Odds Ratio (P-value)	95% CI	
Main Model: the single nearest facility	1.66 (0.03)	(1.04, 2.63)	9,695
Sensitivity Analysis 1: the best facility within 5 km	1.88 (0.05)	(0.99, 3.58)	6,429
Sensitivity Analysis 2: the best facility within 10 km	1.81 (0.03)	(1.06, 3.09)	9,296
Sensitivity Analysis 3: the best facility within 20 km	1.85 (0.10)	(0.87, 3.93)	9,682

*** p<.01, ** p<.05, * p<.1

†Adjusted for: sick child age, sex, type of illness, mother education, household wealth quintile, and the household's rural residence.

For peer review only - http://bmjopen.bmj.com/site/about/guidelines.xhtml

STROBE Statement—Checklist of items that should be included in reports of ***cross-sectional studies***

	Item No	Recommendation	Page/Line number (in the clean version)
**Title and abstract**	1	(*a*) Indicate the study's design with a commonly used term in the title or the abstract	Page 1
		(*b*) Provide in the abstract an informative and balanced summary of what was done and what was found	Page 2-3
**Introduction**			
Background/rationale	2	Explain the scientific background and rationale for the investigation being reported	Page 4-6
Objectives	3	State specific objectives, including any prespecified hypotheses	Page 6 (line 118- 126)
**Methods**			
Study design	4	Present key elements of study design early in the paper	Page 7- 8
Setting	5	Describe the setting, locations, and relevant dates, including periods of recruitment, exposure, follow-up, and data collection	Page 6, line 130- 137
Participants	6	(*a*) Give the eligibility criteria, and the sources and methods of selection of participants	Page 7- 8 and line 265- 287
Variables	7	Clearly define all outcomes, exposures, predictors, potential confounders, and effect modifiers. Give diagnostic criteria, if applicable	Page 8- 10
Data sources/ measurement	8*	For each variable of interest, give sources of data and details of methods of assessment (measurement). Describe comparability of assessment methods if there is more than one group	Page 8- 10
Bias	9	Describe any efforts to address potential sources of bias	Page 11- 12
Study size	10	Explain how the study size was arrived at	Line 265-287
Quantitative variables	11	Explain how quantitative variables were handled in the analyses. If applicable, describe which groupings were chosen and why	Line 169- 222, Line 247- 252
Statistical methods	12	(*a*) Describe all statistical methods, including those used to control for confounding	Line 227 -262
		(*b*) Describe any methods used to examine subgroups and interactions	NA
		(*c*) Explain how missing data were addressed	Line 202- 208
		(*d*) If applicable, describe analytical methods taking account of sampling strategy	Line 196- 202
		(*e*) Describe any sensitivity analyses	Line 253- 259
**Results**			
Participants	13*	(a) Report numbers of individuals at each stage of study—eg numbers potentially eligible, examined for eligibility, confirmed eligible, included in the study, completing follow-up, and analysed	Line 265- 287
		(b) Give reasons for non-participation at each stage	Line 265- 287
		(c) Consider use of a flow diagram	NA
Descriptive data	14*	(a) Give characteristics of study participants (eg	Line 349- 352 &

For peer review only

		demographic, clinical, social) and information on exposures and potential confounders	Table 2
		(b) Indicate number of participants with missing data for each variable of interest	Line 266- 273
Outcome data	15*	Report numbers of outcome events or summary measures	Line 329- 344
Main results	16	(*a*) Give unadjusted estimates and, if applicable, confounder-adjusted estimates and their precision (eg, 95% confidence interval). Make clear which confounders were adjusted for and why they were included	Line 359- 389
		(*b*) Report category boundaries when continuous variables were categorized	NA
		(*c*) If relevant, consider translating estimates of relative risk into absolute risk for a meaningful time period	NA
Other analyses	17	Report other analyses done—eg analyses of subgroups and interactions, and sensitivity analyses	Line 401- 402
**Discussion**			
Key results	18	Summarise key results with reference to study objectives	Line 397- 400
Limitations	19	Discuss limitations of the study, taking into account sources of potential bias or imprecision. Discuss both direction and magnitude of any potential bias	Line 443- 474
Interpretation	20	Give a cautious overall interpretation of results considering objectives, limitations, multiplicity of analyses, results from similar studies, and other relevant evidence	Line 397- 506
Generalisability	21	Discuss the generalisability (external validity) of the study results	Line 489- 500
**Other information**			
Funding	22	Give the source of funding and the role of the funders for the present study and, if applicable, for the original study on which the present article is based	Line 523- 524

*Give information separately for exposed and unexposed groups.

Note: An Explanation and Elaboration article discusses each checklist item and gives methodological background and published examples of transparent reporting. The STROBE checklist is best used in conjunction with this article (freely available on the Web sites of PLoS Medicine at http://www.plosmedicine.org/, Annals of Internal Medicine at http://www.annals.org/, and Epidemiology at http://www.epidem.com/). Information on the STROBE Initiative is available at www.strobe-statement.org.

For peer review only

BMJ Open

Exploring the association between sick child healthcare utilization and health facility quality in Malawi: A Cross-Sectional Study

Journal:	*BMJ Open*
Manuscript ID	bmjopen-2019-029631.R2
Article Type:	Research
Date Submitted by the Author:	24-Jun-2019
Complete List of Authors:	LIU, LINGRUI; Yale University School of Public Health, Health Policy and Management; Global Health Leadership Initiative Yale University<br>Leslie, Hannah; Harvard TH Chan School of Public Health, Department of Global Health and Population<br>Joshua, Martias; Malawi Ministry of Health<br>Kruk, Margaret; Harvard T.H. Chan School of Public Health , Department of Global Health and Population
**Primary Subject Heading**:	Global health
Secondary Subject Heading:	Health services research
Keywords:	health facility quality, sick child healthcare, healthcare utilization, Malawi, sub-Saharan Africa

SCHOLARONE™
Manuscripts

Title

Exploring the association between sick child healthcare utilization and health facility quality in Malawi: A Cross-Sectional Study

Authors

Lingrui Liu[1,2], Hannah H. Leslie[3], Martias Joshua[4], Margaret E. Kruk[3]

Authors' Affiliations

[1]Department of Health Policy and Management, Yale School of Public Health, New Haven, CT, USA
[2]Global Health Leadership Initiative, Yale University, New Haven, CT, USA
[3]Department of Global Health and Population, Harvard T.H. Chan School of Public Health, Boston, MA, USA
[4]Malawi Ministry of Health, Lilongwe, Malawi

Corresponding author:

Lingrui Liu
Department of Health Policy and Management
Yale School of Public Health
New Haven, Connecticut 06510, USA
Lingrui.liu@yale.edu

Word Count (excluding title page, abstract, references, figures and tables) : 4983

For peer review only

For peer review only - http://bmjopen.bmj.com/site/about/guidelines.xhtml

ABSTRACT (word count: 299)

Objective: Increasing the availability of basic healthcare services in low-and-middle-income countries is not sufficient to meet the Sustainable Development Goal target for child survival in high-mortality settings, where healthcare utilization is often inconsistent and quality of care can be poor. We assessed whether poor quality of sick child healthcare in Malawi is associated with low utilization of sick child healthcare.

Design: We measured two elements of quality of sick child healthcare: facility structural readiness and process of care using data from the 2013 Malawi Service Provision Assessment. Overall quality was defined as the average of these metrics. We extracted demographic data from the 2013/2014 Malawi Multiple Indicator Cluster Survey and linked households to nearby facilities using geocodes. We used logistic regression to examine the association of facility quality with utilization of formal health services for children under five suffering diarrhea, fever, or cough/acute respiratory illness, controlling for demographic and socioeconomic characteristics. We conducted sensitivity analyses, modifying the travel distance and population - facility matching criteria.

Setting and Population: 568 facilities were linked with 9,701 children with recent illness symptoms in Malawi, of whom 69% had been brought to a health facility.

Results: Overall, facilities showed gaps in structural quality (62% readiness) and major deficiencies in process quality (33%), for an overall quality score of 48%. Better facility quality was associated with higher odds of utilization of sick child healthcare services (Adjusted odds ratio [AOR]: 1.66, 95% CI:1.04, 2.63), as was structural quality alone (AOR: 1.33, 95% CI:0.95, 1.87). Sensitivity analyses supported the main finding.

Conclusion: Although Malawi's health facilities for curative child care are widely available, quality and utilization of sick child healthcare services are in short supply. Improving facility

For peer review only - http://bmjopen.bmj.com/site/about/guidelines.xhtml

quality may provide a way to encourage higher utilization of healthcare and thereby decrease preventable childhood morbidity and mortality.

Keywords: health facility quality, sick child healthcare, healthcare utilization, Malawi, sub-Saharan Africa

Strengths and limitations of this study

- Using the spatial geocodes, this study linked the health system (2013 Malawi SPA) and the household (2013 Malawi MICS) datasets to investigate the role of quality in sick child healthcare utilization in Malawi.
- This study relied on internationally endorsed guidelines to define quantifiable quality of care measures.
- Multiple sensitivity analyses confirmed the findings.
- However, matching strategy of linking a sick child to a health facility may not completely reflect actual behavior.
- We acknowledged that the MICS survey data on symptoms of illness do not provide sufficient specificity on illness severity and thus were unable to determine that all children with these symptoms in fact require formal healthcare at health facilities.

For peer review only

For peer review only - http://bmjopen.bmj.com/site/about/guidelines.xhtml

INTRODUCTION

The global health community has achieved notable gains in the Millennium Development Goals (MDGs) era. However, elimination of preventable and treatable child mortality remains an urgent global health priority in the coming decade (1, 2). Over the past decade under the umbrella goal of universal health coverage (UHC) (3, 4), health policies have focused on the expansion of coverage of essential health interventions and basic services in sub-Saharan Africa (SSA) and other low-income regions. Due to the rising recognition that, without improving quality of care in the health system, improved access to healthcare alone would not achieve expected health outcomes, the global health community has begun to focus on improvement of healthcare quality (5, 6). Large expansions of health facility networks have been attained in SSA and other low-income regions, and yet, utilization of available resources for care of sick children under five remains low, resulting in inadequate care-seeking for children with diarrhea, malaria or pneumonia (7-10). Malawi, a sub-Saharan African country whose government has declared reduction of preventable children-under-five mortality as a national priority, achieved the MDG targets for improved child health (11). However, healthcare utilization for conditions treatable by the health system is low, as data from the most recent Demographic and Health Survey (DHS) 2015 in Malawi shows (12). Although healthcare facility utilization increased steadily from 2000 to 2015, the proportion of sick children under five with symptoms of acute respiratory infection (ARI), fever, and diarrhea who were taken to a health provider for treatment within 48 hours of symptom onset, remained insufficient (51%, 46%, and 66%, respectively) (7). These utilization patterns occur despite wide availability of child health services (including outpatient curative care, child growth monitoring, and child vaccination) in Malawi's health facilities (12), with a median distance to the nearest health facility of 5 km and an estimated median traveling time of

For peer review only - http://bmjopen.bmj.com/site/about/guidelines.xhtml

75 minutes (13). There is a growing recognition that, besides geographic access, poor quality of care could be a supply-side barrier to UHC, deterring patients from obtaining treatment and influencing family decisions to use or avoid services, which would ultimately impact health outcome gains (14-17).

Although Malawi has achieved substantial improvement in coverage (utilization by those in need) for curative care in children, as estimated from household survey data, quality remains weak (18, 19). Moreover, a recent multi-country study demonstrated that the duration and the content of sick child healthcare service was inadequate (20). Another recent study echoed that care quality in pneumonia diagnosis for a sick child in Malawi is poor, with low guideline adherence to the Integrated Management of Childhood Illness (IMCI) criteria (21). Most existing evaluation studies on the quality of sick child healthcare delivered at health facilities in Malawi have aimed to describe the state of quality but few have tackled the question of whether poor quality dissuades families from using the health system when a child falls ill. In the area of reproductive health, one study suggested a positive association between the perceived quality of reproductive care at the facility last attended and the family utilization of immunization and treatment services for their children at these health facilities, as observed within facilities in Kisumu Municipality in Western Kenya (22). Another recent research from a health intervention program in Ntcheu district in Malawi identified that quality of perinatal care provided is an important predictor of both women's use and satisfaction with such services received (23). Furthermore, earlier literature on bypassing for facility delivery in rural Africa has documented that quality of care is influencing clinic choice (24). Although these studies suggested a positive association between respondent perception of quality and utilization, it would be worthwhile to investigate this relationship at a broader population level (e.g., using national household-

For peer review only - http://bmjopen.bmj.com/site/about/guidelines.xhtml

representative data), as well as through use of the most recent health facility assessment standardized surveys, to explore the relationship.

Policymakers need evidence on what health system attributes (e.g., quality of sick child care) may influence primary care utilization. Malawi is focusing on health sector strategies to improve child health and wellbeing, and has embarked on its second Health Sector Strategic Plan (HSSP 2011-2016), making this an opportune time to seek evidence on the health system factors (e.g., quality) that best promote utilization of sick child healthcare services. In this paper we examine the association between quality of care in health facilities in Malawi and utilization of sick child healthcare by caregivers. This study is one of the few which we are aware that focuses on the contribution of facility quality to utilization of child healthcare services in high-disease-burden settings (25).

METHODS

Study Sample

Malawi is one of the smallest and most densely-populated countries in sub-Saharan Africa, made up of a predominantly young population, of which almost half is aged 15 years or younger, and more than 20% children under five. With a total population of 17 million and a gross domestic product (GDP) per capita of US$1200 in 2015, approximately 52% of Malawi's population lives below the international poverty line ($1.90 per person per day in 2011 purchasing power parity [PPP] dollars), with more than 80% inhabiting rural regions (26). Health facilities that provide child health services in the formal sector of Malawi include hospitals, health centers, clinics, dispensaries, and health posts.

For peer review only - http://bmjopen.bmj.com/site/about/guidelines.xhtml

138 The analysis in this research combined data from multiple sources. To obtain information on the facility quality of child health services, we used the Malawi 2013-14 Service Provision Assessment (SPA), a comprehensive census of all formal-sector health facilities conducted by the Demographic and Health Surveys (DHS) Program (12). SPA is a standardized cross-sectional survey of public and private health facilities, which includes a facility-level audit, clinical observation protocols, patient exit interviews, and healthcare provider interviews. The health facility audit was conducted in each health facility visited using a standardized inventory questionnaire. At least one health worker was interviewed in a sampled health facility. Interviewers also observed a sample of patient-provider clinical care service interaction processes, as well as interviewing patients observed receiving care upon their exit from the health facility. The SPA survey does not report or examine clinical outcomes. In this study, we examined the quality of out-patient sick child curative care delivered at health facilities in Malawi, excluding child vaccination services and child growth monitoring services. We limited our sample of facilities to those facilities (including hospitals, health centers, health posts, dispensaries) which provided outpatient sick child curative care services.

153 To obtain household information and care-seeking for children under five years of age, we used the 2013/2014 Malawi Multiple Indicator Cluster (27). Malawi 2013 MICS is a household survey conducted to assess health outcomes among a nationally-representative sample of the population, employing a multi-stage sampling strategy. Enumeration areas (EAs) were sampled within the strata of district and urban versus rural location, and then households were identified within EAs. With a systematic sample of 25 households drawn in each sample cluster, a total sample of 1140 EAs and 28,479 households were selected for the Malawi 2013 MICS. We limited our sample of households to those in which caregivers reported children under five years

For peer review only - http://bmjopen.bmj.com/site/about/guidelines.xhtml

of age who had an episode of diarrhea, symptoms of acute respiratory infection (ARI), or fever during the two weeks prior to the survey. Although these three specific medical conditions cannot cover the full range of illnesses of children under five, malaria, diarrhea, and pneumonia remain leading causes of death among children under age five and are the most consistent indicators for child disease burden across the globe, with the vast majority occurring in LMICs (28). These conditions combined are relevant in epidemiological predictions and represent demand for sick child healthcare services in Malawi.

Measure of quality of child health services delivered at health facilities in Malawi

To date, there has not been a single uniform set of measures on quality definition and metrics (15, 29). The Institute of Medicine report *Crossing the Quality Chasm* identified six dimensions to measure quality: safe, effective, patient-centered, timely, efficient, and equitable (30). We consolidated these dimensions with Donabedian's (1980) quality of care framework of structure, process and outcome (31). The structure elements describe the characteristics of the environment in which healthcare is provided, which exist before the care takes place. The structural inputs of a health system indicate the capability and serviceability of a health system under which care occurs (32). Process features include two key components: technical interventions (appropriate delivery of clinical procedures following clinical guidelines, e.g., WHO Integrated Management of Childhood Illness [IMCI] guidelines in child curative care services) and inter-personal interactions between users and a healthcare system. Outcomes refer to the final consequences of healthcare, such as the under-five mortality rate (32). In this study, available data did not specify patient outcomes for the children experiencing recent illness.

For peer review only - http://bmjopen.bmj.com/site/about/guidelines.xhtml

183 Using Donabedian's framework, we constructed an index of structural quality based on the facility audit and an index of process quality based on the observation of healthcare delivered. We reviewed the WHO Service Availability and Readiness Assessment (SARA) to identify relevant structural quality indicators and the WHO Integrated Management of Childhood Illness (IMCI) for expected clinical actions and matched these to items available in the SPA survey. A total of 29 items on three domains were identified matching the SARA health facility readiness items (general readiness and readiness for the specific child curative care service) to assess the structure quality, using data extracted from the SPA health facility audits: (1) infrastructure (e.g., water, electricity, ambulance, etc.); (2) equipment, essential supplies and medications (e.g., gloves, sharps, medications storage, daily computer updates, etc.); (3) staffing and management (e.g., supervision provided, staff training, etc.). Moreover, 18 indicators were identified matching the IMCI items to assess the observed clinical care process quality. This process quality index covers assessment of clinical history, routine examination, and counseling on danger signs. We first averaged structural indicators within facility. We then averaged process indicators within observation. Further, we averaged these clinical observations for sick child visits within each facility to obtain the facility level process quality score, using a rescaled weight for each clinical observation to reflect the sampling probability of patients within facilities. We further averaged the facility-level structure and process quality score to arrive at a facility-level overall quality score to measure average performance on all indicators. For each quality index, indicators were averaged to provide a facility summary score from 0 to 1. Multiple imputation was applied to address the missingness of individual indicators for facilities without sick child observations (up to 19%; see Appendix Table 1 for details), using the R Amelia package (33). Considering the multiple imputation assumption that missingness is random conditional on the covariates, we

For peer review only - http://bmjopen.bmj.com/site/about/guidelines.xhtml

206 included four covariates in our imputation: facility type (e.g., central hospital, district hospital,
207 health centers, etc.), facility managing authority (e.g., government, private, NGO, mission/faith-
208 based, etc.), districts, and urban/rural.

209

210 **Sick child care utilization**

211 Utilizing sick child healthcare was defined as a binary indicator that children with
212 diarrhea, fever, or symptoms of acute respiratory illness (ARI) sought curative care at formal
213 health facilities. Following the MICS report in identifying which response options were
214 considered as formal care and being consistent with the facility types surveyed in SPA, we
215 consider health facilities including hospitals, health centers, clinics, health posts, and
216 dispensaries, and did not include other sources of informal care (e.g. traditional healers or shops).

217

218 **Covariates**

219 We obtained data from the MICS on household socioeconomic status (household wealth
220 index, urban/rural residence), and demographics of caretaker and ill child (child's age and
221 gender, mother's education level) as well as child illness type. The household wealth index was
222 calculated following standard procedures for the DHS and classified into quintiles by the MICS.

223

224 **Patient and public involvement**

225 Patients and the public were not involved in the research design or planning of this study.

226

227 **Statistical analysis**

228 We obtained the spatial location of all enumeration areas (EAs) for the MICS from the

For peer review only

For peer review only - http://bmjopen.bmj.com/site/about/guidelines.xhtml

2008 Malawi census data. Each sick child was linked to the geographic centroid of the associated household's EA using EA codes provided by the Malawi National Statistical Office. SPA data include exact location of each facility. There was limited empirical evidence from Malawi regarding where children were taken to seek treatment for their illness. In this study, rather than assessing bypassing, we focused on whether having a high quality facility nearby promotes household utilization of sick child health services. We matched each sick child and his/her household to the single nearest facility, based on travel distance by road to the EA centroid of the sick child's household. To calculate the geographic distance, we executed four steps: first identified the closet 8 facilities providing sick child healthcare by using the geocoordinates for facilities (exact location) and household clusters (centroid); second, the road distance was calculated between cluster centroid and each facility based on the Google Maps API, including linear distance to nearest road where coordinates are not directly on a road (road type is not incorporated in this calculation); third, we replaced road distance with linear distance if road distance can not be calculated (e.g., if there is no road on an island) or if road distance is less than linear distance; lastly, we identified the facility with minimum distance to the cluster. Steps 1, 3, and 4 were executed in Stata, and Step 2 was run in Python 3.6.1. Based on prior studies suggesting that household distance to nearest health facility in Malawi is rarely greater than 50 km (34-36), we excluded children whose nearest facility was over 50 km away.

Descriptive analyses of facility quality for sick child healthcare were first performed. We used logistic regression analyses of utilization of sick child healthcare on the quality index and then adjusted for the covariates of interest. We used clustered standard errors to account for the non-independence of observations within EAs. To understand which element of the overall

For peer review only - http://bmjopen.bmj.com/site/about/guidelines.xhtml

quality served best as a predictor for household utilization of sick child healthcare, we separated overall facility quality into structural and process quality.

We further conducted a series of sensitivity analyses to understand the robustness of our results. To understand whether the best performing facility may be more influential than the nearest facility, as long as it's still relatively accessible, we matched the household with the best performing facility within the buffer zone of a 5-km radius (direct distance from the household's EA centroid). Additionally, to test the sensitivity of catchment area definitions, we continued using the best performing facility to match with the households, but modified the original 5-km buffer zones to 10-km and 20-km direct distance radius from the household's EA centroid.

Statistical analyses were run in Stata (version 14.1), mapping was done using QGIS Version 2.18 (Free Software Foundation, Massachusetts), and geographic distances (e.g., road traveling distances) were calculated based on Google Maps using Python 3.6.1.

RESULTS

Participants

In the 2013 SPA, 977 of a total of 1,066 surveyed health facilities (response rate: 92%) completed the assessment. Among these 977 facilities, 920 facilities (94%) offered sick child health services. Among these 920 facilities, 746 facilities had observations of sick child healthcare; process quality indicators were imputed for the 174 facilities that offer sick child services but did not have any observations of care. Completeness of each indicator is shown in Appendix Table 1. There was no missingness for infrastructure, but minimal for equipment, essential supplies and medications, substantial for supervision (up to 14%), and moderate for process quality (up to 19%).

For peer review only - http://bmjopen.bmj.com/site/about/guidelines.xhtml

The 2013 MICS dataset included 18,981 children under five years of age with completed caretaker interviews (response rate: 98%). Among these responding households, 52% of children (n=9,811) were reported by their caretakers as having symptoms of diarrhea (n=4,419, 45%), ARI (n=1,438, 15%), or fever (n=7,118, 73%). Among the 9,811 children who had illnesses, 6,679 children (68%) sought care at facilities (hospitals, health centers/clinics, or health posts/dispensaries).

In our main matching strategy, 110 of the 9,811 sick children were dropped due to the EAs for which locations are not available. Then, each of the 9701 sick children was matched to the child's single nearest facility of the 920 health facilities providing sick child care based on road traveling distance to the EA centroid of the sick child's household. In this step, 352 health facilities were dropped. Therefore, our main matching strategy yielded an analytical sample of 568 health facilities providing sick child healthcare services and 9,701 children who were reported by their caregivers as having illness of diarrhea, fever, or ARI. A total of 8,363 (86%) sick child consultations were actually observed in these selected facilities.

Descriptive Data

Table 1 detailed characteristics of health facilities in our main analytic sample. Among the health facilities that were included in the main analytic sample, health centers (57%) and clinics (26%) were the most common. The bulk of health facilities had clinical officers as the highest level of provider present (79%). The number of facilities located in rural areas was about four-fold those in urban settings, while about half were managed by government authority. Figure 1 shows the geographic distribution of all 2013 SPA health facilities and the ones

For peer review only - http://bmjopen.bmj.com/site/about/guidelines.xhtml

included in our analytic sample, the 2013 MICS enumeration areas, as well as the population density in Malawi.

Figure 1 to be inserted here.

For peer review only

For peer review only - http://bmjopen.bmj.com/site/about/guidelines.xhtml

Table 1. Facility characteristics and quality performance on sick child healthcare services in the analytic sample (Main Model: n=568) [£]

	**All facilities (568)**	
	n or mean	% or SD
Rural[§]	445	78.4
Urban	123	21.6
Public[¶]	300	53.0
Private	268	47.0
Facility type		
Hospital	75	13.2
Health center	323	56.9
Clinic	145	25.5
Health post	3	0.5
Other (dispensaries)	22	3.9
Highest clinician on site		
Medical doctor	60	10.6
Registered nurse	8	1.4
Enrolled nurse	38	6.7
Assistant medical officer	9	1.6
Clinical officer	448	78.9
Other health professional	5	0.9
Overall Quality Performance (mean, SD)		
Structural quality	0.62	0.14
Process quality	0.33	0.14
Overall quality	0.48	0.10

For peer review only

For peer review only - http://bmjopen.bmj.com/site/about/guidelines.xhtml

£ In the main model (n-568), the analysis was restricted to facilities offering sick child healthcare that matched (within 50km) to a household sampled in the MICS with a recently ill child.

.

§ Facility is in rural area.

¶ Facility is managed by government authority.

Figures 2 and 3 detail the facility performance on a structural and process quality index, respectively. The average structural quality score for health facilities in Malawi providing sick child curative care services was 0.62 (SD: 0.14, range: 0.20- 0.97) and the average process quality score was 0.33 (SD: 0.14, range: 0.04- 0.78). The average overall quality score was 0.48 (SD: 0.10, range: 0.19- 0.90). Facilities were commonly equipped with basic infrastructure, such as client waiting rooms and general facility cleanliness, while they still lacked light sources, electricity, toilet, ambulance, and computer or internet access. Health facilities achieved high level performance on essential supplies and medication readiness, while about forty percent (40%) had sick child health service room infection control supplies. Within the staffing and management domain, nearly all facilities reported employing some form of supervision, while a modest number performed well in routine quality assurance or else received inadequate staff training on IMCI child health services. Reporting client opinions was extremely rare. With the exception of high percentages in process quality indicators achieved for fever, coughing, and temperature examination, all other indicators performed relatively modestly. The poorest indicator was providing counseling and examination for danger signs, edema, maternal-infant transmission of HIV, and ear pain.

Figure 2 to be inserted here.

Figure 3 to be inserted here.

Table 2 provides characteristics of sick children in the analytic sample, of whom, 6,679 children (69%) sought care at facilities during their illness, while 3,022 (31%) did not seek care. The average age of sick children was 29 months (SD: 16 months), with 16% younger than one

For peer review only - http://bmjopen.bmj.com/site/about/guidelines.xhtml

350 year old. Half (50%) of the sick children were girls. The bulk of sick children had a perceived symptom of fever (64%), with ARI the least common (15%). The average road traveling distance to the nearest facility was about 5.8 km (SD: 4.7 km; median: 4.7 km).

For peer review only

For peer review only - http://bmjopen.bmj.com/site/about/guidelines.xhtml

353 **Table 2. Characteristics of sick child in the analytic sample (Main model: n=12,258)**

	Total sick children (n=9,701)		Sought for care at facilities during illness (n= 6,679)	No care-seeking during illness (n= 3,022)
**Variable**	**n**	**%§**	**n(%¶)**	**n(%¶)**
**Child age (in months)**				
**≤ 2 months**	162	1.7	88(54.3%)	74(45.7%)
**2 months- 1 year**	1,537	15.8	1052(68.4%)	485(31.6%)
**1 year - 5 years**	8,002	82.5	5539(69.2%)	2463(30.8%)
**Child sex**				
**Male**	4,870	50.2	3420(70.2%)	1450(29.8%)
**Female**	4,831	49.8	3259(67.5%)	1572(32.5%)
**Child sickness type**				
**Diarrhea**	2,097	21.6	1284(61.2%)	813(38.8%)
**Fever**	6,185	63.8	4352(70.4%)	1833(29.6%)
**ARI**	1,419	14.6	1043(73.5%)	376(26.5%)
**Mother's education**				

For peer review only - http://bmjopen.bmj.com/site/about/guidelines.xhtml

**None**	1,287	13.2	817(63.5%)	470(36.5%)
**Primary**	7,016	72.3	4828(68.8%)	2188(31.2%)
**Secondary or above**	1,392	14.3	1031(74.1%)	361(25.9%)
**Household wealth quintile**				
**Q1 (poorest)**	2,380	24.5	1605(67.4%)	775(32.6%)
**Q2**	2,227	23	1515(68.0%)	712(32.0%)
**Q3**	2,140	22.1	1472(68.8%)	668(31.2%)
**Q4**	1,703	17.6	1205(70.8%)	498(29.2%)
**Q5 (richest)**	1,251	13	882(70.5%)	369(29.5%)
**Road Traveling Distance (km) to nearest facility mean (SD, median)**	5.77(4.7, 4.8)		5.66(4.7, 4.7)	6.00(4.7, 4.9)

§ Column percentages.
¶ Row percentages.
£ n=9,701, number of children who were perceived as sick in last two weeks by their caretakers that plausibly matched with their households' single nearest sick child health service facility in SPA. In the main model (n-568), the analysis was restricted to facilities offering sick child healthcare that matched (within 50km) to a household sampled in the MICS with a recently ill child.

For peer review only

For peer review only - http://bmjopen.bmj.com/site/about/guidelines.xhtml

359 **Main findings**

360 Table 3 show results of the multivariable logistic regression models of the association
361 between quality of care and sick child healthcare service utilization in Malawi health facilities.
362 Model 1 and Model 4 represent the base and fully-specified models, respectively. Models 2 and
363 3 represent models adjusting for individual factors. Model 5 represents the fully specified model
364 with the quality index separated into structural inputs and process quality.

365 The overall quality index of structural and process quality was a significant predicator of
366 utilizing formal health facilities for sick child healthcare services in Malawi. In the fully
367 specified model (Model 4), the odds of utilizing formal health facilities increases with increasing
368 quality (AOR: 1.66, 95% CI: 1.04, 2.63). The magnitude of the effect increases slightly when
369 adjusting for the control variables, and remains significant in all models (Model 1-4). When
370 separating the overall quality index into structural inputs and the process quality in the full
371 specified model (Model 5), structural quality was a significant predictor (AOR: 1.33, 95% CI:
372 0.95, 1.87) while process quality was positively but not significantly associated with utilization
373 (AOR: 1.25, 95% CI: 0.91, 1.72). The overall quality remained as a significant predicator for
374 utilizing health facilities for sick child healthcare in the three sensitivity analyses (SA) shown in
375 Appendix Table 2. These SA findings supported the association between facility quality and sick
376 child healthcare utilization even when using a large catchment area.

377 A child's type of illness and mother's education were found to be significant individual-
378 level predictors of sick child healthcare utilization. For children's illness type, in the fully
379 specified model (Model 4) the results suggest higher odds of utilizing health facilities for
380 children with a reported fever (AOR: 1.17, 95% CI: 1.03, 1.34) and symptoms of ARI
381 (AOR:1.78, 95% CI: 1.53, 2.06), compared to those who were perceived to have diarrhea. This

For peer review only - http://bmjopen.bmj.com/site/about/guidelines.xhtml

382 indicates that the type of childhood illness (i.e., diarrhea, fever, or ARI) is associated with
383 motivating caregivers to utilize health facilities. For mother's education, the results suggest a
384 gradient with increasing odds of utilizing health facilities as the level of the mother's education
385 increases. Compared to mothers who did not have primary education, mothers who had primary
386 education and those had secondary or higher education had 30% (AOR: 1.30, 95% CI: 1.13,
387 1.49) and 65% (AOR:1.65, 95% CI: 1.38, 1.97) higher odds of utilizing health facilities,
388 respectively, after controlling for other factors.

For peer review only

For peer review only - http://bmjopen.bmj.com/site/about/guidelines.xhtml

389 **Table 3. Regression results for the association between sick child healthcare utilization and the overall quality (structural and**
390 **process quality) of health service facility in Malawi.** £

**Main Models**	(1)		(2)		(3)		(4)		(5)	
VARIABLES	Odds ratio (P-value)	95% CI¶	Adjusted odds ratio (P-value)	95% CI	Adjusted odds ratio (P-value)	95% CI	Adjusted odds ratio (P-value)	95% CI	Adjusted odds ratio (P-value)	95% CI
Overall quality	1.53(0.08)	(0.96, 2.43)	1.61 (0.05)	(1.01, 2.56)	1.67 (0.03)	(1.05, 2.65)	1.66(0.03)	(1.04, 2.63)	-	
Structural quality			-		-		-		1.33(0.10)	(0.95, 1.87)
Process quality			-		-		-		1.25(0.17)	(0.91, 1.72)
Child age										
(Ref: ≤ 2 months)										
2 months- 1 year			0.99(0.90)	(0.88, 1.12)	1.00 (1.00)	(0.89, 1.13)	1.00(1.00)	(0.89, 1.13)	1.00(1.00)	(0.89, 1.13)
1 year- 5 years			1.98(0.00)	(1.45, 2.72)	1.99 (0.00)	(1.45, 2.73)	1.99(0.00)	(1.45, 2.73)	1.99(0.00)	(1.46, 2.73)
Child sex										
(Ref: Male)										
Female			0.88 (0.00)	(0.80, 0.95)	0.88 (0.00)	(0.80, 0.96)	0.88(0.00)	(0.80, 0.96)	0.88(0.00)	(0.80, 0.96)
Child sickness type										
(Ref: diarrhea)										
Fever			1.17 (0.02)	(1.02, 1.33)	1.17 (0.02)	(1.03, 1.34)	1.17(0.02)	(1.03, 1.34)	1.17(0.02)	(1.03, 1.34)

For peer review only - http://bmjopen.bmj.com/site/about/guidelines.xhtml

ARI			1.77 (0.00)	(1.53, 2.06)	1.78 (0.00)	(1.54, 2.06)	1.78(0.00)	(1.53, 2.06)	1.79(0.00)	(1.53, 2.06)
Mother education										
(Ref: No education)										
Primary					1.29 (0.00)	(1.12, 1.49)	1.29(0.00)	(1.13, 1.49)	1.30(0.00)	(1.13, 1.49)
Secondary or Higher					1.65 (0.00)	(1.38, 1.97)	1.65(0.00)	(1.38, 1.97)	1.65(0.00)	(1.38, 1.97)
Household Wealth Quintile (Ref: Q1 poorest)										
Q2					0.91 (0.24)	(0.07)	0.92(0.38)	(0.77, 1.10)	0.92(0.38)	(0.77, 1.10)
Q3					0.96 (0.60)	(0.08)	0.98(0.82)	(0.82, 1.17)	0.98(0.82)	(0.82, 1.17)
Q4					0.99 (0.87)	(0.08)	1.01(0.92)	(0.84, 1.21)	1.01(0.91)	(0.84, 1.21)
Q5					1.00 (0.96)	(0.08)	1.02(0.84)	(0.85, 1.22)	1.01(0.83)	(0.85, 1.22)
Household Rural Residence							0.95(0.57)	(0.80, 1.13)	0.95(0.56)	(0.80, 1.13)
Observations	9,701		9,701		9,695		9,695		9,695	

*** p<.01, ** p<.05, * p<.1

£ In the main model (n-568), the analysis was restricted to facilities offering sick child healthcare that matched (within 50km) to a household sampled in the MICS with a recently ill child.

For peer review only - http://bmjopen.bmj.com/site/about/guidelines.xhtml

For peer review only

[¶] 95% CI: 95% Confidence Intervals

For peer review only

For peer review only - http://bmjopen.bmj.com/site/about/guidelines.xhtml

DISCUSSION

In this paper, we sought to determine the influence of facility-based quality of healthcare on care-seeking behaviors of Malawian parents faced with childhood illness. We found that facility quality is an important predictor of parental decision-making regarding care-seeking for illnesses such as diarrhea, fever, and ARI among children under five years, using national data from Malawi. Our sensitivity analyses varying the catchment areas consistently supported this association.

We found that about 69% of surveyed caregivers for sick children in our analytic sample utilized facility-based healthcare services in Malawi, reflecting a similar prevalence of care-seeking reported by the most recent two Malawi DHS surveys (7, 37). Although Malawi has been one of the top three countries with highest prevalence of care-seeking for children with reported fever, diarrhea, or symptoms of ARI in African maternal and child health (MCH) priority countries, care-seeking for sick under-five children was still not optimal given the target of ending preventable deaths of under five children by 2030 in Malawi Health Sector Strategic Plan (HSSP).

Our findings confirmed past research showing major gaps in service readiness and provider competence in sick child healthcare. Like this work, others have noted poor provider performance, with fewer than half of clinical actions completed (e.g., taking patient history, examination, and counseling) (18, 20). Moreover, guideline adherence was low, with a high rate of missed diagnosis among children (four out of every five children) with pneumonia (38).

Our work advances on prior research in several ways. Past studies have largely focused on other factors in determining a caregiver's appropriate care-seeking for childhood illness, such as socio-demographic and household characteristics, social cultural factors, geographic access,

For peer review only - http://bmjopen.bmj.com/site/about/guidelines.xhtml

travel time, health facility fees, insurance, health worker densities, etc (39-43). Most prior studies on quality care for sick child healthcare services were specific evaluations of quality improvement interventions, such as IMCI community health worker programs (44-46). Few studies have examined the relationship of quality and healthcare utilization on a national scale with regard to childhood illness in LMICs, with only one publication from Kenya attempting to construct the relationship between patient-perceived quality and the low attendance at the maternal and child health services using local district data.

When disaggregating our quality measure into structural and process components, we found that structural readiness (facility infrastructure, equipment, and staff) was a significant predictor of utilization, while process quality (adherence to medical guidelines) was positively but not significantly predictive of utilization. This finding suggests that individuals (caregivers) may weigh visible health facility characteristics (such as presence of client waiting room, general facility cleanness, equipment, drugs, etc.) more than they weigh the actual clinical care service experience. Because of the asymmetry of information between providers and patients in the healthcare market, patients do not have full knowledge about what constitutes good quality; in this case, they may not be fully aware of the recommended components of clinical assessment for their child (47).

This study had several strengths. First, the availability of exact spatial location data of the SPA health facilities and all EAs for the MICS from the 2008 Malawi census data provided a unique opportunity to examine health system and population data together in concert to answer a policy relevant question. By contrast, DHS household locations are displaced up to 5km in rural areas, which precludes accurate matching to nearest facility. Second, we relied on WHO SARA

For peer review only - http://bmjopen.bmj.com/site/about/guidelines.xhtml

and IMCI guidelines to define quality measures. Furthermore, multiple sensitivity analyses confirmed that the findings were consistent across different facility matching specifications.

However, this study has several limitations. First, this study is based on cross-sectional data and is therefore unable to address causal claims. Second, given the available Malawi healthcare utilization data (2013 MICS), we chose the most clinically relevant questions (variables) to indicate the demand by under-five children with sickness of diarrhea, malaria, or pneumonia. Our study focused on understanding the influence of quality on caregivers' decisions to utilize health facilities for their sick child. However, no patient outcome data were available, which prevented investigation of the linkage between facility quality and patient outcomes. Third, due to the data limitations, we extracted the quality index items available from the Malawi SPA dataset with reliance on the WHO SARA and IMCI, and therefore this analysis focused on specific health facility characteristics and interviewer-observed clinical quality service, which however do not assess individual perceptions of healthcare quality, nor address the patient-provider interpersonal quality of care. Additionally, we acknowledged the common challenge of classifying childhood illness based on survey data in LMICs, (48, 49) thereby our research was unable to capture the sufficient specificity on illness severity and identify all children with these symptoms who actually require formal healthcare at health facilities. Furthermore, in our multiple imputation strategy, we assumed that missingness of the health facility which provided sick child healthcare but had 0 observations within a stratum like rural dispensaries is random. Our assumption was not that the missingness of these observations is randomly distributed across hospitals and dispensaries. Therefore, we included four covariates (facility type, facility managing authority, districts, and rural/urban) in the multiple imputation. Moreover, in the real world, caretaker decisions in seeking care for a sick child, as well as where to seek healthcare,

For peer review only - http://bmjopen.bmj.com/site/about/guidelines.xhtml

can be influenced by their perception of the quality of child health services at facilities recently visited. Our study used the facility-based patient data that captures the nearest facility to the patient's household and we were not able to identify the previous facility which the patient visited. Given literature from other nations with comparable settings, in their first action, caregivers would seek healthcare at the nearest facility upon recognizing the child's symptom; however, they would choose to bypass their nearest facility when it lacked diagnostic equipment, drugs, and skilled health workers, or had poor services. (22, 50-52) . In this study, we were not able to examine the influence of quality of the previous facility in relation to the caregiver's next health services facility choice, although these types of associations have been beneficial in prior studies in developed countries (53). Thus, the matching strategy of linking a sick child to a health facility may not completely reflect actual behavior.

Policy Implications

Our findings have several implications for policy and future research. Our study provides strong and direct empirical evidence that better quality health facilities are associated with increased healthcare utilization for childhood illness, which, if care is sufficiently competent, can improve odds of survival from treatable conditions. As our findings suggested, the government of Malawi may consider the improvement of the health facility structure, including equipment, essential supplies, drug storage and availability, room cleanness and infection control. Visible improvements are most likely to attract caregivers in utilization of the health facilities when they perceive childhood illness. However, beyond driving utilization, provider clinical competence needs to improve as well if visits are to be converted into better health. Given the low level of provider performance we and others have documented, this will require structure system reforms,

For peer review only - http://bmjopen.bmj.com/site/about/guidelines.xhtml

potentially including updating pre-service education and re-organizing where healthcare is delivered for maximum gains (6).

Future research is needed to validate and extend these findings in other country settings. Malawi has been a leader in sub-Saharan Africa, demonstrating strong political will to implement evidence-based interventions that can improve maternal and child health. The availability of the geocoded health facilities and population data enabled us to match the health system facility survey data and the population data in. Other countries could take similar measures to permit matching of health system and household data to obtain better insights in how health systems influence health and care seeking. These measures would aid in policy determinations, to evaluate whether services supplied by the health system can actually meet with the demands of the population, and quality of care can satisfy the population's needs and promote patient care-seeking behaviors that reduce preventable deaths. In addition, the facility quality index needs to be validated in different country settings, encompassing higher mortality burdens and different health system capacities, to strengthen the generalizability of the results.

As an important component of human capital, health can contribute positively to a nation's economic development (54). At the same time, achieving an excellent state of health is an intrinsic part of the goals of social development, as well as an essential factor in an individual's well-being. Good quality healthcare is thus both an intrinsic good that can promote health outcomes as well as a driver of utilization; to achieve these ends both structures and processes of care need to improve.

For peer review only

For peer review only - http://bmjopen.bmj.com/site/about/guidelines.xhtml

Acknowledgements

Earlier versions of this paper were presented during several seminars at the Harvard Global Health Institute, the Harvard T.H. Chan School of Public Health, and the Fifth Global Symposium on Health Systems Research, where valuable comments were received from seminar participants, including Stéphane Verguet, Michael Reich and Sara Singer. The authors thank Dennis Lee and Fei Carnes for assistance with geographic linking. The authors also acknowledge Humphreys Nsona for his input on the initial study idea and assistance with the geospatial data.

Contributors: MEK conceptualized the study. HHL and LL curated data. LL conducted the formal analysis. HHL contributed to study design. LL visualized the data results and wrote the original draft. LL, HHL, MJ, and MEK reviewed and contributed to the editing of the manuscript. All authors approved the final manuscript submitted.

Funding: This research received no specific grant from any funding agency in the public, commercial or not-for-profit sectors.

Competing Interests Statement: None declared.

Patient Consent: Patients and the public were not involved in the research design or planning of the study therefore the patient consent was not required.

Ethical approval: The Harvard University Human Research Protection Program deemed this analysis of secondary data exempt from human subject review.

Data Sharing Statement: SPA data files are available from the DHS Program (http://dhsprogram.com/whatwedo/survey/survey-display-424.cfm). MICS survey are available from UNICEF (http://mics.unicef.org/surveys); cluster sampling details were provided on request from the Malawi National Statistical Office.

For peer review only

For peer review only - http://bmjopen.bmj.com/site/about/guidelines.xhtml

References

1. The United Nations Millennium Development Goals. [cited 2017 September 20]. Available from: http://www.un.org/millenniumgoals/.
2. The United Nations Sustainable Development Goals. Available from: http://www.un.org/sustainabledevelopment/sustainable-development-goals/.
3. World Health Organization. World health report 2013: Research for universal health coverage. 2013.
4. United Nations General Assembly. Ga/11326. [cited 2017 October 20]. Available from: http://www.un.org/en/ga/67/resolutions.shtml.
5. Berwick DM, Kelley E, Kruk ME, et al. Three global health-care quality reports in 2018. The Lancet. 2018;392(10143):194-5.
6. Kruk ME, Gage AD, Arsenault C, et al. High-quality health systems in the sustainable development goals era: Time for a revolution. The Lancet Global Health. 2018;6(11):e1196-e252.
7. National Statistical Office (NSO) [Malawi] and ICF. Malawi demographic and health survey 2015-16. Zomba, Malawi, and Rockville, Maryland, USA. NSO and ICF: 2017.
8. Central Statistical Agency (CSA) [Ethiopia] and ICF. Ethiopia demographic and health survey 2016. Addis Ababa, Ethiopia, and Rockville, Maryland, USA: CSA and ICF: 2016.
9. Ministry of Health CD, Gender, Elderly and Children (MoHCDGEC) [Tanzania Mainland], Ministry of Health (MoH) [Zanzibar], National Bureau of Statistics (NBS), Office of the Chief Government Statistician (OCGS), and ICF. Tanzania demographic and health survey and malaria indicator survey (tdhs-mis) 2015-16. Dar es Salaam, Tanzania, and Rockville, Maryland, USA: MoHCDGEC, MoH, NBS, OCGS, and ICF: 2016.
10. Kenya National Bureau of Statistics MoHK, National AIDS Control Council/Kenya, Kenya Medical Research Institute, National Council for Population and Development/Kenya, and ICF International. Kenya demographic and health survey 2014. Rockville, MD, USA: Kenya National Bureau of Statistics, Ministry of Health/Kenya, National AIDS Control Council/Kenya, Kenya Medical Research Institute, National Council for Population and Development/Kenya, and ICF International: 2015.
11. United Nations Development Porgramme (UNDP). 2014 millennium development goal report for malawi. United Nations Development Programme, New York: 2014.
12. The Demographic and Health Surveys. Malawi service provision assessment (spa) 2013-14.
13. National Statistical Office/Malawi and Macro International. Malawi demographic and health survey 1992. Zomba, Malawi: National Statistical Office/Malawi and Macro International: 1994.
14. Sobel HL, Huntington D, Temmerman M. Quality at the centre of universal health coverage. Health Policy and Planning. 2015;31(4):547-9.
15. Hanefeld J, Powell-Jackson T, Balabanova D. Understanding and measuring quality of care: Dealing with complexity. Bulletin of the World Health Organization. 2017;95(5):368.
16. Kruk ME, Larson E, Twum-Danso NA. Time for a quality revolution in global health. The Lancet Global Health. 2016;4(9):e594-e6.
17. Abera Abaerei A, Ncayiyana J, Levin J. Health-care utilization and associated factors in gauteng province, south africa. Global health action. 2017;10(1):1305765.
18. Leslie HH, Ndiaye Y, Kruk ME. Effective coverage of primary care services in eight high-mortality countries. BMJ Global Health. 2017;2(3):e000424.

For peer review only - http://bmjopen.bmj.com/site/about/guidelines.xhtml

19. Gera T, Shah D, Garner P, et al. Integrated management of childhood illness (imci) strategy for children under five: Effects on death, service utilisation and illness. Cochrane Database of Systematic Reviews. 2012(9).
20. Kruk ME, Gage AD, Mbaruku GM, et al. Content of care in 15,000 sick child consultations in nine lower-income countries. Health services research. 2018.
21. Uwemedimo OT, Lewis TP, Essien EA, et al. Distribution and determinants of pneumonia diagnosis using integrated management of childhood illness guidelines: A nationally representative study in malawi. BMJ global health. 2018;3(2):e000506.
22. Audo M, Ferguson A, Njoroge P. Quality of health care and its effects in the utilisation of maternal and child health services in kenya. East African Medical Journal. 2005;82(11).
23. Creanga AA, Gullo S, Kuhlmann AKS, et al. Is quality of care a key predictor of perinatal health care utilization and patient satisfaction in malawi? BMC pregnancy and childbirth. 2017;17(1):150.
24. Kruk ME, Mbaruku G, McCord CW, et al. Bypassing primary care facilities for childbirth: A population-based study in rural tanzania. Health Policy and Planning. 2009;24(4):279-88.
25. Gage AD, Leslie HH, Bitton A, et al. Assessing the quality of primary care in haiti. Bulletin of the World Health Organization. 2017;95(3):182.
26. World bank data indicators [Internet]. [cited March 03, 2017]. Available from: http://data.worldbank.org/.
27. National Statistical Office. Malawi mdg endline survey 2014. Zomba, Malawi: National Statistical Office; 2015
28. UNICEF, WHO, World Bank, UN-DESA Population Division. Levels and trends in child mortality report 2017. New York: United Nations Children's Fund, 2017.
29. Kruk ME, Pate M, Mullan Z. Introducing the lancet global health commission on high-quality health systems in the sdg era. The Lancet Global Health. 2017;5(5):e480-e1.
30. The Institute of Medicine. Crossing the quality chasm: A new health system for the 21st century. 2001
31. Donabedian A. Evaluating the quality of medical care. Milbank Quarterly. 2005;83(4):691-729.
32. Campbell SM, Roland MO, Buetow SA. Defining quality of care. Social science & medicine. 2000;51(11):1611-25.
33. Honaker J, King G, Blackwell M. Amelia ii: A program for missing data. Journal of statistical software. 2011;45(7):1-47.
34. Ewing VL, Lalloo DG, Phiri KS, et al. Seasonal and geographic differences in treatment-seeking and household cost of febrile illness among children in malawi. Malaria Journal. 2011;10(1):32.
35. Karra M, Fink G, Canning D. Facility distance and child mortality: A multi-country study of health facility access, service utilization, and child health outcomes. International Journal of Epidemiology. 2016;46(3):817-26.
36. Malawi National Statistical Office. Malawi demographic and health survey 1992 National Statistical Office, Zomba, Malawi, 1992.
37. National Statistical Office (NSO) and ICF Macro. Malawi demographic and health survey 2010. Zomba, Malawi, and Calverton, Maryland, USA: NSO and ICF Macro: 2011.

For peer review only - http://bmjopen.bmj.com/site/about/guidelines.xhtml

38. Omolara T. Uwemedimo, Todd P. Lewis, Elsie A. Essien, et al. Distribution and determinants of pneumonia diagnosis using integrated management of childhood illness guidelines: A nationally representative study in malawi. BMJ Global Health. Forthcoming.
39. Abdulkadir MB, Abdulkadir ZA. A cross-sectional survey of parental care-seeking behavior for febrile illness among under-five children in nigeria. Alexandria Journal of Medicine. 2017;53(1):85-91.
40. Noordam AC, Carvajal-Velez L, Sharkey AB, et al. Correction: Care seeking behaviour for children with suspected pneumonia in countries in sub-saharan africa with high pneumonia mortality. PLoS One. 2015;10(4):e0126997.
41. Shaw B, Amouzou A, Miller NP, et al. A qualitative exploration of care-seeking pathways for sick children in the rural oromia region of ethiopia. BMC health services research. 2017;17(1):184.
42. Chibwana AI, Mathanga DP, Chinkhumba J, et al. Socio-cultural predictors of health-seeking behaviour for febrile under-five children in mwanza-neno district, malawi. Malaria journal. 2009;8(1):219.
43. Hjortsberg C. Why do the sick not utilise health care? The case of zambia. Health economics. 2003;12(9):755-70.
44. Cardemil CV, Gilroy KE, Callaghan-Koru JA, et al. Comparison of methods for assessing quality of care for community case management of sick children: An application with community health workers in malawi. The American journal of tropical medicine and hygiene. 2012;87(5_Suppl):127-36.
45. Miller NP, Amouzou A, Tafesse M, et al. Integrated community case management of childhood illness in ethiopia: Implementation strength and quality of care. The American journal of tropical medicine and hygiene. 2014;91(2):424-34.
46. Nsona H, Mtimuni A, Daelmans B, et al. Scaling up integrated community case management of childhood illness: Update from malawi. The American journal of tropical medicine and hygiene. 2012;87(5_Suppl):54-60.
47. Arneill AB, Devlin AS. Perceived quality of care: The influence of the waiting room environment. Journal of Environmental Psychology. 2002;22(4):345-60.
48. Munos MK, Stanton CK, Bryce J. Improving coverage measurement for reproductive, maternal, neonatal and child health: Gaps and opportunities. Journal of global health. 2017;7(1).
49. Arnold F, Khan SM. Perspectives and implications of the improving coverage measurement core group's validation studies for household surveys. Journal of global health. 2018;8(1).
50. Kruk ME, Hermosilla S, Larson E, et al. Bypassing primary care clinics for childbirth: A cross-sectional study in the pwani region, united republic of tanzania. Bulletin of the World Health Organization. 2014;92(4):246-53.
51. Kahabuka C, Kvåle G, Moland KM, et al. Why caretakers bypass primary health care facilities for child care-a case from rural tanzania. BMC health services research. 2011;11(1):315.
52. Lohela TJ, Campbell OM, Gabrysch S. Distance to care, facility delivery and early neonatal mortality in malawi and zambia. PLoS One. 2012;7(12):e52110.
53. Jung K, Feldman R, Scanlon D. Where would you go for your next hospitalization? Journal of Health Economics. 2011;30(4):832-41.
54. Bloom DE, Canning D, Sevilla J. The effect of health on economic growth: A production function approach. World development. 2004;32(1):1-13.

For peer review only - http://bmjopen.bmj.com/site/about/guidelines.xhtml

Figure 1. Distribution of health facilities in Malawi.

Figure 2. Performance on facility structural quality index: percentage of facilities with key resources and services (n=568).

Figure 3. Performance on facility process quality index: percentage of facilities with key resources and services (n=568).

For peer review only

For peer review only - http://bmjopen.bmj.com/site/about/guidelines.xhtml

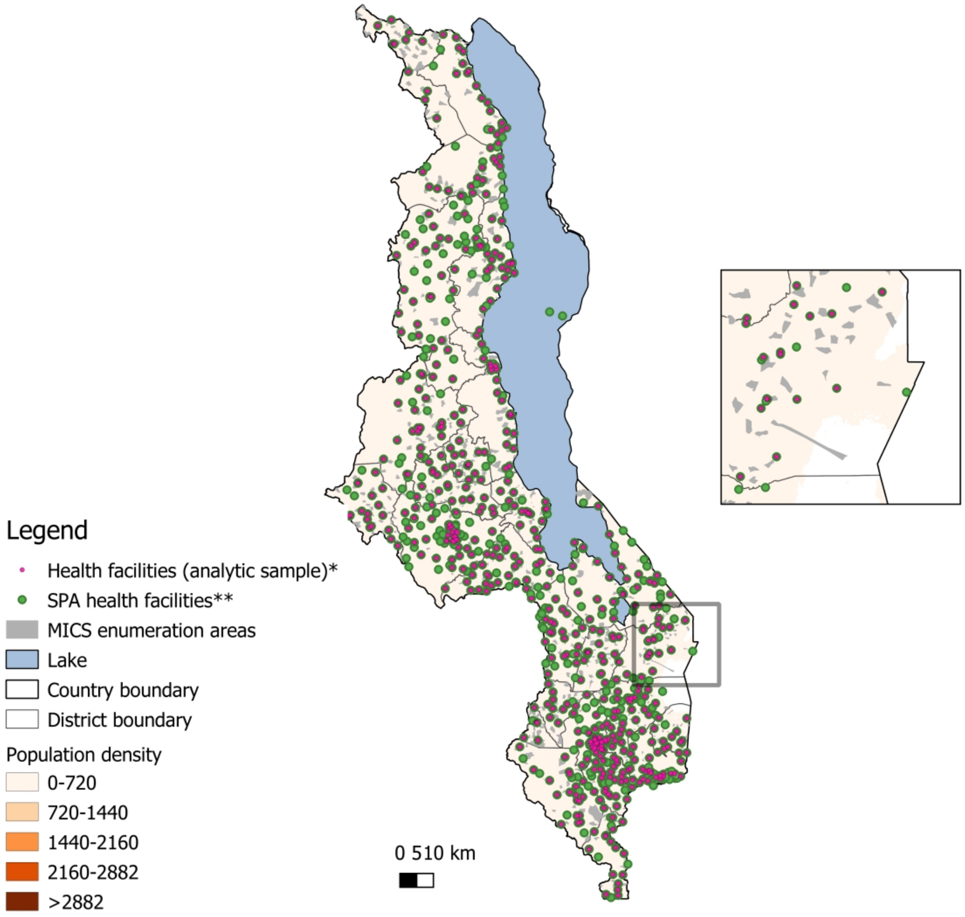

Figure 1. Distribution of health facilities in Malawi. Data source: the map was created using the following data sources: (1) Population density: Worldpop.org (open access) https://www.worldpop.org/geodata/summary?id=123, WorldPop. 2017. Malawi 100m Population, Version 2. University of Southampton. DOI: 10.5258/SOTON/WP00538. (2) MICS enumeration areas: Malawi National Statistical Office. (3) Malawi shapefile and health facility location: The DHS Program. Notes: *Analytic sample (n=568). In the main model, the analysis was restricted to facilities offering sick child healthcare that matched (within 50 km) to a household sampled in the MICS with a recently ill child. **Malawi SPA health facilities (n=977): the health facilities completed the assessment in Malawi 2013 SPA.

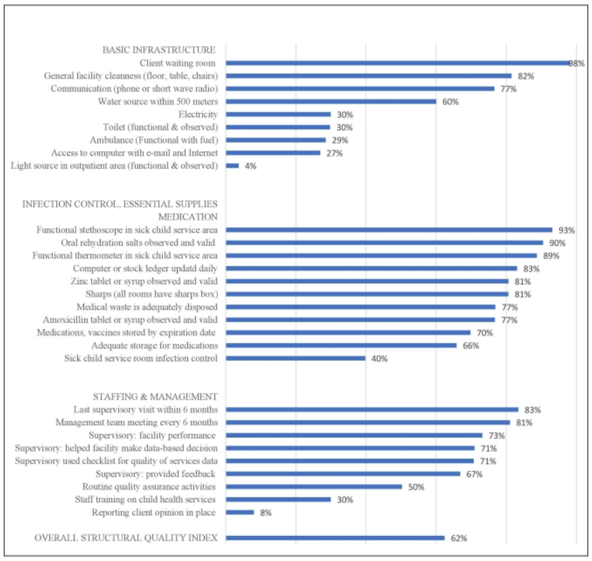

Figure 2. Performance on facility structural quality index: percentage of facilities with key resources and services (n=568).

338x190mm (300 x 300 DPI)

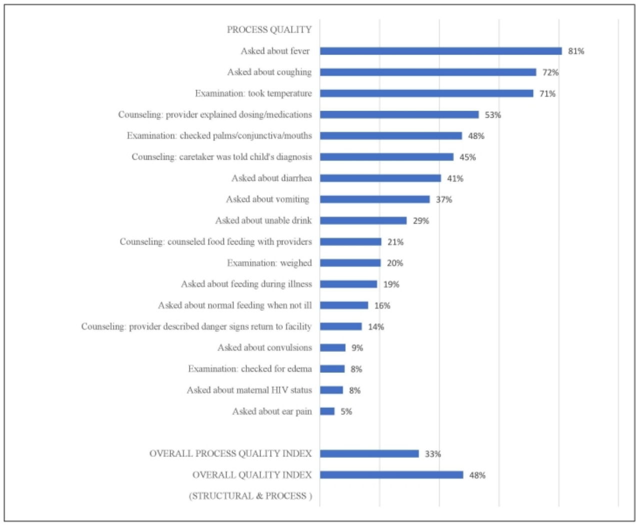

Figure 3. Performance on facility process quality index: percentage of facilities with key resources and services (n=568).

338x190mm (300 x 300 DPI)

Appendix Table 1: Completeness of indicators for facilities† (n=920) and observations

Variable	% Missing	% Completed
Water (improved water source round within 500 meters)	0	100
Ambulance (Functional with fuel)	0	100
Electricity	0	100
Communication (phone or short wave radio)	0	100
Toilet (functional & observed)	0	100
General facility cleanness (floor, table, chairs)	0	100
Client waiting room	0	100
Access to computer with e-mail and Internet	0	100
Light source (functional & observed)	0	100
Sick child health service room infection control	0	100
Sharps (all rooms have sharps box)	0.33	99.67
Medical waste is adequately disposed	0.43	99.57
Functional thermometer in sick child health service area	0	100
Functional stethoscope in sick child health service area	0	100
Oral rehydration salts in pharmacy or sick child health service area	0	100
Amoxicillin tablet or syrup observed and valid	0.65	99.35
Zinc tablet or syrup observed and valid	0.65	99.35
Medications, vaccines stored according to expiration date	11.96	88.04
Adequate storage for medications	0	100
Computer or stock ledger updated daily	0	100
Staff training on child health services	0	100
Last supervisory visit within 6 months	0	100
Management team meeting every 6 months	0.65	99.35
Supervisory used checklist for quality of health services data	13.70	86.30

For peer review only - http://bmjopen.bmj.com/site/about/guidelines.xhtml

Supervisory: facility performance	13.70	86.30
Supervisory: helped facility make data-based decision	13.70	86.30
Supervisory: provided feedback	13.70	86.30
Reporting client opinion in place	1.20	98.8
Routine quality assurance activities	0.87	99.13
Asked about vomiting	18.91	81.09
Asked about unable to drink	18.91	81.09
Asked about convulsions	18.91	81.09
Asked about normal feeding when not ill	18.91	81.09
Asked about maternal HIV status	18.91	81.09
Asked about feeding during illness	18.91	81.09
Asked about fever	18.91	81.09
Asked about ear pain	18.91	81.09
Asked about diarrhea	18.91	81.09
Asked about coughing	18.91	81.09
Examination: weighed	18.91	81.09
Examination: took temperature	18.91	81.09
Examination: checked palms/conjunctiva/mouths	18.91	81.09
Examination: checked for edema	18.91	81.09
Counseling: provider explained dosing/medications	19.24	80.76
Counseling: counseled food feeding with providers	18.91	81.09
Counseling: caretaker was told child's diagnosis	18.91	81.09
Counseling: provider described danger signs return to facility	18.91	81.09

† A total of 920 health facilities from 2013 Malawi SPA that provided child curative care was included in this study.

For peer review only - http://bmjopen.bmj.com/site/about/guidelines.xhtml

Appendix Table 2. Sensitivity analyses model results for the association between sick child healthcare utilization and the overall quality (structural and process quality) of health service facility in Malawi.

**Model**	**Adjusted results** †		**N**
	Odds Ratio (P-value)	95% CI	
Main Model: the single nearest facility	1.66 (0.03)	(1.04, 2.63)	9,695
Sensitivity Analysis 1: the best facility within 5 km	1.88 (0.05)	(0.99, 3.58)	6,429
Sensitivity Analysis 2: the best facility within 10 km	1.81 (0.03)	(1.06, 3.09)	9,296
Sensitivity Analysis 3: the best facility within 20 km	1.85 (0.10)	(0.87, 3.93)	9,682

*** p<.01, ** p<.05, * p<.1

†Adjusted for: sick child age, sex, type of illness, mother education, household wealth quintile, and the household's rural residence.

For peer review only - http://bmjopen.bmj.com/site/about/guidelines.xhtml

STROBE Statement—Checklist of items that should be included in reports of ***cross-sectional studies***

	Item No	Recommendation	Page/Line number (in the clean version)
**Title and abstract**	1	(*a*) Indicate the study's design with a commonly used term in the title or the abstract	Page 1
		(*b*) Provide in the abstract an informative and balanced summary of what was done and what was found	Page 2-3
**Introduction**			
Background/rationale	2	Explain the scientific background and rationale for the investigation being reported	Page 4-6
Objectives	3	State specific objectives, including any prespecified hypotheses	Page 6 (line 118- 126)
**Methods**			
Study design	4	Present key elements of study design early in the paper	Page 7- 8
Setting	5	Describe the setting, locations, and relevant dates, including periods of recruitment, exposure, follow-up, and data collection	Page 6, line 130- 137
Participants	6	(*a*) Give the eligibility criteria, and the sources and methods of selection of participants	Page 7- 8 and line 265- 287
Variables	7	Clearly define all outcomes, exposures, predictors, potential confounders, and effect modifiers. Give diagnostic criteria, if applicable	Page 8- 10
Data sources/ measurement	8*	For each variable of interest, give sources of data and details of methods of assessment (measurement). Describe comparability of assessment methods if there is more than one group	Page 8- 10
Bias	9	Describe any efforts to address potential sources of bias	Page 11- 12
Study size	10	Explain how the study size was arrived at	Line 265-287
Quantitative variables	11	Explain how quantitative variables were handled in the analyses. If applicable, describe which groupings were chosen and why	Line 169- 222, Line 247- 252
Statistical methods	12	(*a*) Describe all statistical methods, including those used to control for confounding	Line 227 -262
		(*b*) Describe any methods used to examine subgroups and interactions	NA
		(*c*) Explain how missing data were addressed	Line 202- 208
		(*d*) If applicable, describe analytical methods taking account of sampling strategy	Line 196- 202
		(*e*) Describe any sensitivity analyses	Line 253- 259
**Results**			
Participants	13*	(a) Report numbers of individuals at each stage of study—eg numbers potentially eligible, examined for eligibility, confirmed eligible, included in the study, completing follow-up, and analysed	Line 265- 287
		(b) Give reasons for non-participation at each stage	Line 265- 287
		(c) Consider use of a flow diagram	NA
Descriptive data	14*	(a) Give characteristics of study participants (eg	Line 349- 352 &

For peer review only

		demographic, clinical, social) and information on exposures and potential confounders	Table 2
		(b) Indicate number of participants with missing data for each variable of interest	Line 266- 273
Outcome data	15*	Report numbers of outcome events or summary measures	Line 329- 344
Main results	16	(*a*) Give unadjusted estimates and, if applicable, confounder-adjusted estimates and their precision (eg, 95% confidence interval). Make clear which confounders were adjusted for and why they were included	Line 359- 389
		(*b*) Report category boundaries when continuous variables were categorized	NA
		(*c*) If relevant, consider translating estimates of relative risk into absolute risk for a meaningful time period	NA
Other analyses	17	Report other analyses done—eg analyses of subgroups and interactions, and sensitivity analyses	Line 401- 402
**Discussion**			
Key results	18	Summarise key results with reference to study objectives	Line 397- 400
Limitations	19	Discuss limitations of the study, taking into account sources of potential bias or imprecision. Discuss both direction and magnitude of any potential bias	Line 443- 474
Interpretation	20	Give a cautious overall interpretation of results considering objectives, limitations, multiplicity of analyses, results from similar studies, and other relevant evidence	Line 397- 506
Generalisability	21	Discuss the generalisability (external validity) of the study results	Line 489- 500
**Other information**			
Funding	22	Give the source of funding and the role of the funders for the present study and, if applicable, for the original study on which the present article is based	Line 523- 524

*Give information separately for exposed and unexposed groups.

Note: An Explanation and Elaboration article discusses each checklist item and gives methodological background and published examples of transparent reporting. The STROBE checklist is best used in conjunction with this article (freely available on the Web sites of PLoS Medicine at http://www.plosmedicine.org/, Annals of Internal Medicine at http://www.annals.org/, and Epidemiology at http://www.epidem.com/). Information on the STROBE Initiative is available at www.strobe-statement.org.

For peer review only
